# Recyclable Heterogeneous Chitosan Supported Copper Catalyst for Silyl Conjugate Addition to α,β-Unsaturated Acceptors in Water

**DOI:** 10.3390/polym10040385

**Published:** 2018-04-01

**Authors:** Lei Zhu, Bojie Li, Shan Wang, Wei Wang, Liansheng Wang, Liang Ding, Caiqin Qin

**Affiliations:** 1 School of Chemistry and Materials Science, Hubei Engineering University, Hubei Collaborative Innovation Center of Conversion and Utilization for Biomass Resources, Xiaogan 432000, China; libojie0614@hotmail.com (B.L.); smallcoral@live.cn (S.W.); 15327403696@163.com (W.W.); wangls@hbeu.edu.cn (L.W.); 2 Department of Polymer and Composite Material, School of Materials Engineering, Yancheng Institute of Technology, Yancheng 224051, China

**Keywords:** chitosan supported copper, heterogeneous catalyst, organosilicon compound, easily recyclable

## Abstract

The first example of an environmentally-benign chitosan supported copper catalyzed conjugate silylation of α,β-unsaturated acceptors was accomplished in water under mild conditions. This protocol provides an efficient pathway to achieve an important class of β-silyl carbonyl compounds and the desired products were obtained in good to excellent yields. Gram-scale synthesis and easy transformation of obtained β-silyl products were also been demonstrated. Remarkably, this chitosan supported copper catalyst can be easily recycled and reused six times without any significant decrease of catalytic activity. The advantages of this newly developed method include operational simplicity, good functional group tolerance, scale-up ability, ready availability, and easy recyclability of catalyst.

## 1. Introduction

The development of methods for the synthesis of organosilicon compounds has attracted intense attention because of their myriad uses in organic synthesis [1,2] as well as their potential applications in materials science [3] and biological chemistry [4,5]. Traditionally, most β-silyl carbonyl compounds are prepared via conjugate addition process by using stoichiometric amounts of silyl metal reagents [6,7,8]. Besides, the methods that utilized a catalytic amount of metal in the presence of silylzincates [9,10,11] or silylcuprates [12] were also developed for further improvement of efficiency. In recent years, the utilization of Suginome’s silylboron reagent [13], dimethylphenylsilyl pinacolatoboronate (**1**), provided an alternative and effective strategy to directly install the dimethylphenylsilyl moiety to α,β-unsaturated acceptors. Although, Rh(I) [14,15,16,17], Cu(I) [18,19,20], and metal-free NHC [21] catalysts were successfully applied for activating the Si–B bond of Suginome’s reagent, Cu(II)-catalysis is still in high demand because its protocol is much more convenient with lower cost. Until now, only a few methods based on Cu(II) catalysis have been disclosed for this transformation. For example, our group [22] and Santos [23] independently reported that Cu_2_(OH)_2_CO_3_ and CuSO_4_ respectively could catalyze the silylation of α,β-unsaturated compounds in the presence of a base. Kobayashi and co-workers [24] carried out this reaction in an enantioselective manner by using a chiral complex obtained from Cu(acac)_2_ and a chiral 2,2’-bipyridine ligand. In consideration of limited examples of Cu(II) catalysis and environmental impact, it is necessary to develop a green, efficient, and much milder strategy using Cu(II) catalysis to obtain functionalized organosilicon compounds bearing a carbonyl moiety. Furthermore, the recycling and reuse of Cu(II) catalyst still remains a challenge due to the deactivation or decomposition of non-immobilized Cu(II) salts. 

Transition metal catalysts immobilized on a heterogeneous support played an important role in discovering unique reactivities and selectivities different from homogeneous systems [25,26]. The observed heterogeneous catalyst displays several advantages such as easy isolation, operational simplicity, environmental compatibility and remarkable reusability. Various supports—including magnetic materials [27], zeolites [28,29], silica [30], and polymers [31]—have been adopted for the immobilization of transition metals. Recently, chitosan (CS) has received extensive interest due to its non-toxicity, biodegradability, and reasonable ability of chelation [32,33]. It was proven that chitosan supported metal complexes were efficient to catalyze C–C [34,35,36,37,38,39], C–N [40], C–O [41], and C–S [42] bonds forming reactions. Meanwhile, we reported that chitosan supported copper is capable of catalyzing the transfer of pinacolatoboron moiety to α,β-unsaturated carbonyls, leading to the formation of C–B bond [43]. However, to the best of our knowledge, very few examples describing the C–Si formation in chitosan supported heterogeneous system were reported previously and the topic still represents a challenge.

With our continuous efforts in exploring applications of chitosan supported metal catalysts [22,43], we were interested in developing a green and mild protocol for synthesis of useful organosilicon compounds. Hence, we herein report a simple and easily available chitosan supported Cu(II) material as a highly reactive and recyclable catalyst for the desired β-silyl conjugate additions of α,β-unsaturated acceptors. 

## 2. Materials and Methods 

### 2.1. Materials

Chitosan (degree of acetylation = 5%; *M*_W_: 10,000–50,000, determined by GPC) was purchased from Aladdin (Shanghai, China), dimethylphenylsilyl pinacolatoboronate (CAS: 185990-03-8) was purchased from Energy Chemical (Shanghai, China) and Cu(II) salts were purchased from J&K (Beijing, China). All α,β-unsaturated acceptors, bases were obtained commercially from Energy Chemical (Shanghai, China) and used without further purification. Chitosan supported copper catalysts CS@CuSO_4_, CS@Cu(OAc)_2_, and CS@Cu(acac)_2_ were prepared according to the procedures reported [42].

### 2.2. Analytical Methods

Nuclear magnetic resonance (NMR) spectra were recorded on a Bruker Avance III 600 MHz spectrometer (Karlsruhe, Germany), operating at 600 for ^1^H and 150 MHz for ^13^C NMR in CDCl_3_ unless otherwise noted. CDCl_3_ is served as the internal standard (δ = 7.26 ppm) for ^1^H NMR and (δ = 77.0 ppm) for ^13^C NMR. Data for ^1^H-NMR is reported as follows: chemical shift (ppm, scale), multiplicity (s = singlet, d = doublet, t = triplet, q = quartet, m = multiplet and/or multiplet resonances, br = broad), coupling constant (Hz), and integration. Data for ^13^C NMR is reported in terms of chemical shift (ppm, scale), multiplicity, and coupling constant (Hz). Flash column chromatographic purification of products was accomplished using forced-flow chromatography on silica gel (200–300 mesh). The weight percentage and metal leaching of copper were determined by inductively coupled plasma-optical emission spectroscopy (ICP-OES) (PerkinElmer, Waltham, MA, USA) analysis. The copper loading of CS@CuSO_4_, CS@Cu(OAc)_2_, and CS@Cu(acac)_2_ were found to be 1.85, 1.42, and 1.50 mmol/g, respectively.

### 2.3. General Procedure for Preparation of Chitosan Supported Copper Catalyst

Chitosan (5 g) was suspended in 100 mL of water. Copper salts (1 g) was added to this suspension and the mixture was stirred for 3 h. The catalyst was separated using a centrifuge (5000 rpm, 10 min), and dried under vacuum at 50 °C.

### 2.4. General Procedure for the Sample Preparation for ICP Analysis to Determine Catalyst Loading

Chitosan supported copper catalyst (~20 mg) was placed in a clean test tube and heated with H_2_SO_4_ (1 mL) at 200 °C. After 30 min, several drops of concentrated HNO_3_ were added carefully and the tube was shaken occasionally. HNO_3_ was continuously added until a clear solution was obtained and excess amount of HNO_3_ was allowed to evaporate under heating. After the solution was cooled to room temperature, 1 mL of aqua regia was added carefully. Effervescence of gas was observed and the solution become clearer. The solution was then transferred to a volumetric flask and made up to 50 mL with water which was submitted for ICP analysis.

### 2.5. General Procedure for the Sample Preparation for ICP Analysis to Determine Metal Leaching

After the reaction was finished, the reaction mixture was filtered. The filtrate obtained was concentrated and diluted with 10 mL of THF. Then 50% *v*/*v* of the crude THF solution (5 mL) was then passed through a membrane filter (0.25 or 0.45 μm) into a clean test tube. After evaporation of solvent, the solid obtained in the test tube was heated to 200 °C and 1.0 mL of concentrated H_2_SO_4_ was added. Following similar procedure described above, concentrated HNO_3_ were added at regular intervals until the resulting solution was clear. After the solution was cooled to room temperature, 1 mL of aqua regia was added carefully. Effervescence of gas was observed and the solution become clearer. The solution was then transferred to a volumetric flask and made up to 50 mL with water which was submitted for ICP analysis.

### 2.6. General Procedure for CS@Cu-Catalyzed Silylation of α,β-Unsaturated Acceptors in Water

CS@Cu(acac)_2_ (10.0 mg, 5 mol % Cu loading) and 4-picoline (3.3 mg, 6 mol %) were mixed in water (2 mL). The mixture was stirred for 1 h at room temperature, followed by successive addition of substrate (**2**) (0.3 mmol) and PhMe_2_Si-B(pin) (**1**) (94.4 mg, 0.36 mmol). After stirring for 12 h at room temperature, the reaction mixture was filtered and the filtrate was extracted with EtOAc (20 mL × 3). Combined organic layers were dried over anhydrous Na_2_SO_4_. After being concentrated under reduced pressure, the crude mixture was purified by flash column chromatography (200–300 mesh silica gel, petroleum ether/EtOAc = 10:1~20:1) to afford the desired product **3**.

### 2.7. General Procedure for Gram-Scale Synthesis of **3a**

CS@Cu(acac)_2_ (150 mg, 5 mol % Cu loading) and 4-picoline (49.5 mg, 6 mol %) were mixed in water (30 mL). The mixture was stirred for 1 h at room temperature, followed by successive addition of chalcone (**2a**) (0.94 g, 4.5 mmol) and PhMe_2_Si-B(pin) (**1**) (1.42 g, 5.4 mmol). After stirring for 12 h at room temperature, the reaction mixture was filtered and the filtrate was extracted with EtOAc (30 mL × 3). Combined organic layers were dried over anhydrous Na_2_SO_4_. After being concentrated under reduced pressure, the crude mixture was purified by flash column chromatography (200–300 mesh silica gel, petroleum ether/EtOAc = 10:1) to afford the desired product **3a**.

### 2.8. Recycling and Reuse of CS@Cu Catalyst

In order to demonstrate the recyclability of CS@Cu(acac)_2_ catalyst, the silyl conjugate addition was repeated six times with the same sample. The initial amount of catalyst was 50.0 mg (5 mol % Cu loading). Reactions were carried out for 12 h. After the reaction, catalyst was filtered off, washed by EtOAc and water, and then dried for 4 h at 60 °C before next run. The recovery rate of the catalyst at the time of reusing is about 92%.

## 3. Results and Discussion

For the initial investigation, we tested the silyl conjugate addition of PhMe_2_SiB(pin) (**1**) to chalcone (**2a**) catalyzed by an array of chitosan supported copper catalysts in water (Table 1, entries 1–3). To our delight, CS@CuSO_4_, CS@Cu(OAc)_2_, and CS@Cu(acac)_2_ were all able to catalyze this transformation to obtain desired β-silyl product in 30%, 22%, and 41% yield respectively. Encouraged by this observation, we turned to screen different bases, including inorganic bases and a series of pyridine derivatives to further improve the conversion (Table 1, entries 4–9). It was found that the reactivity obviously increased in a more basic aqueous solution when sodium carbonate or potassium carbonate were used as base (Table 1, entries 4 and 5). However, 2,2’-bipyridine (**B1**) and its analog (**B2**) resulted in no effect to the reaction system, probably due to their poor dispersability in water (Table 1, entries 6 and 7). Furthermore, we had previously disclosed that 2,2’-bipyridine was effective to activate Si-B bond in the presence of a non-ionic surfactant [22]. Gratifyingly, significant improvements were achieved when pyridine (**B3**) and 4-picoline (**B4**) were used as additives (Table 1, entries 8 and 9). 4-Picoline is favorable for this reaction because it may perform the roles of the ligand for copper and Brønsted base for activating water molecules [23]. In the absence of catalyst, the reaction did not proceed at all (Table 1, entry 10). Subsequently, several aprotic solvents were examined (Table 1, entries 11–14), upon using dichloromethane, tetrahydrofuran, diethylether and toluene as solvents, product **3a** was not observed due to the lack of proton source. Protic solvents such as methanol and ethanol only resulted in very low conversion because of their poor ability to provide proton (Table 1, entries 15 and 16). Notably, it was feasible to reduce the catalyst loading to half without compromising on yield (Table 1, entry 17). In addition, the reaction atmosphere did not affect the reactivity as illustrated (Table 1, entry 18). Therefore, the optimized reaction conditions were determined to run the reaction at room temperature in H_2_O with 6 mol % 4-picoline, using CS@Cu(acac)_2_ as catalyst (Table 1, entry 9).

**Figure 1:**
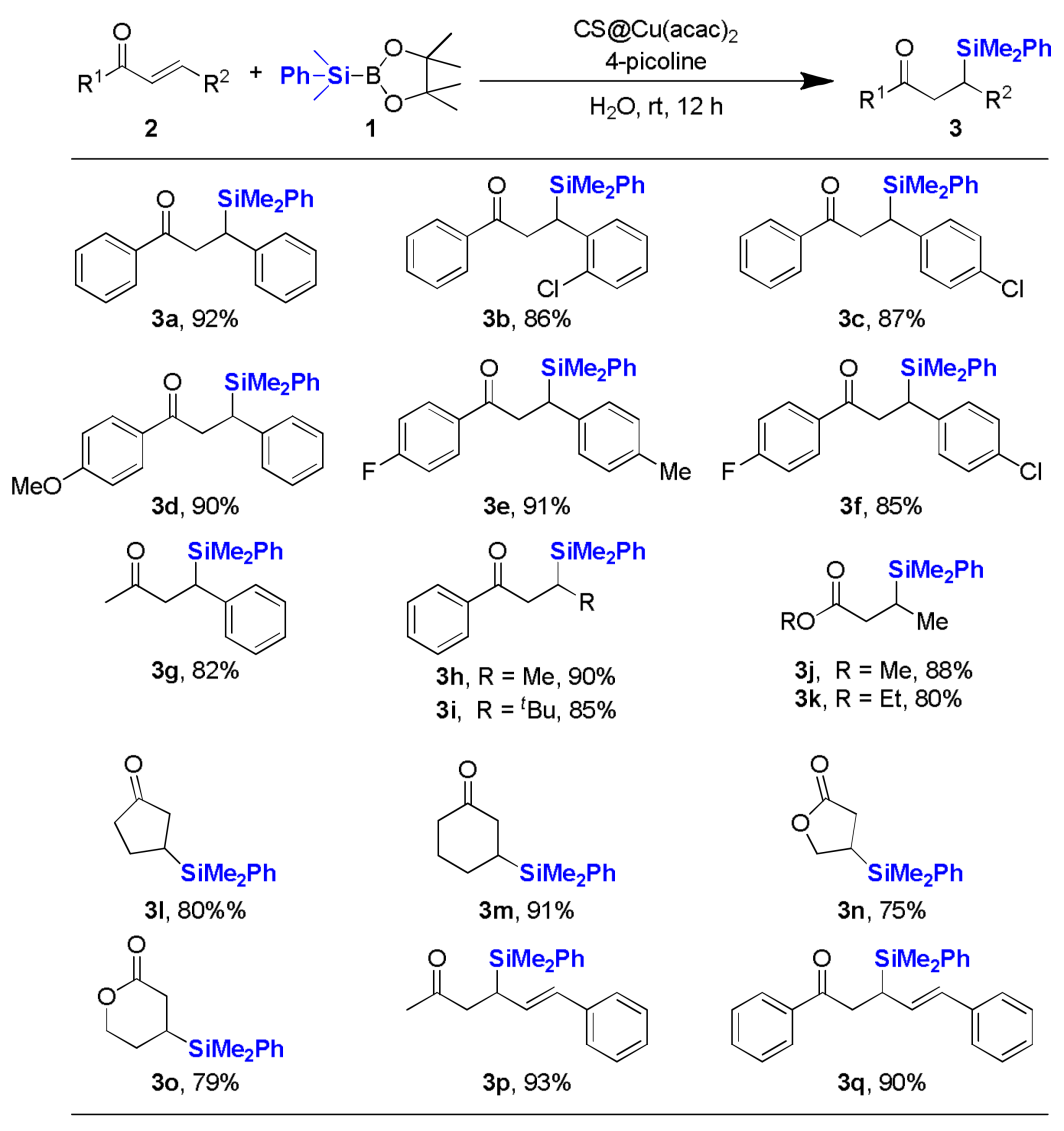
Substrate scope of α,β-unsaturated acceptors ^a,b^. ^a^ Reaction conditions: substrate **2** (0.3 mmol), Me_2_PhSi-B(pin) **1** (1.2 equiv.), CS@Cu(acac)_2_ (5 mol % Cu loading), 4-picoline (6 mol %), H_2_O (2 mL), room temperature, air, 12 h; ^b^ Isolated yields were listed.

With the optimized conditions in hand, the substrate scope of α,β-unsaturated acceptors was surveyed and the results were summarized in Figure 1. Chalcone derivatives bearing a chloro-substituent at *ortho*- or *para*-position both proceeded smoothly to give corresponding products in good yields (**3b**,**3c**) (**3b**–**3q** are in Supplementary Materials). Electron-donating group as well as the electron-withdrawing one was tolerated in this kind of silylation process (**3d**). Next, di-substituted chalcones were also found to be suitable substrates besides mono-substituted derivatives (**3e**,**3f**). Silylation of α,β-unsaturated ketones having aromatic moieties on one side while aliphatic ones on the other, all performed well under the optimized conditions (**3g**,**3h**), even with sterically congested substrate (**3i**). It is worthy to note that great improvements were achieved for methyl- and ethyl-esters by using our newly developed strategy, compared to the previous report (**3j**,**3k**) [23]. Not only acyclic α,β-unsaturated carbonyls, but also cyclic ketones were applicable and exhibited comparable reactivities (**3l**,**3m**). Remarkably, lactones which are common motifs of many natural products could also be reacted with **1** to yield β-silyl products in 75% and 79%, respectively (**3n**,**3o**). Notably, 1,4-addition products were obtained exclusively without the formation of 1,6-addition byproducts when α,β,γ,δ-unsaturated dienones were used as substrates (**3p**,**3q**). The allylsilane products **3p** and **3q** could be further employed as a useful synthon in the Hosomi–Sakurai reaction [44]. Therefore, it was demonstrated that a wide range of α,β-unsaturated acceptors could be silylated effectively which were catalyzed by chitosan supported copper catalyst with 4-picoline as base.

**Scheme 1:**
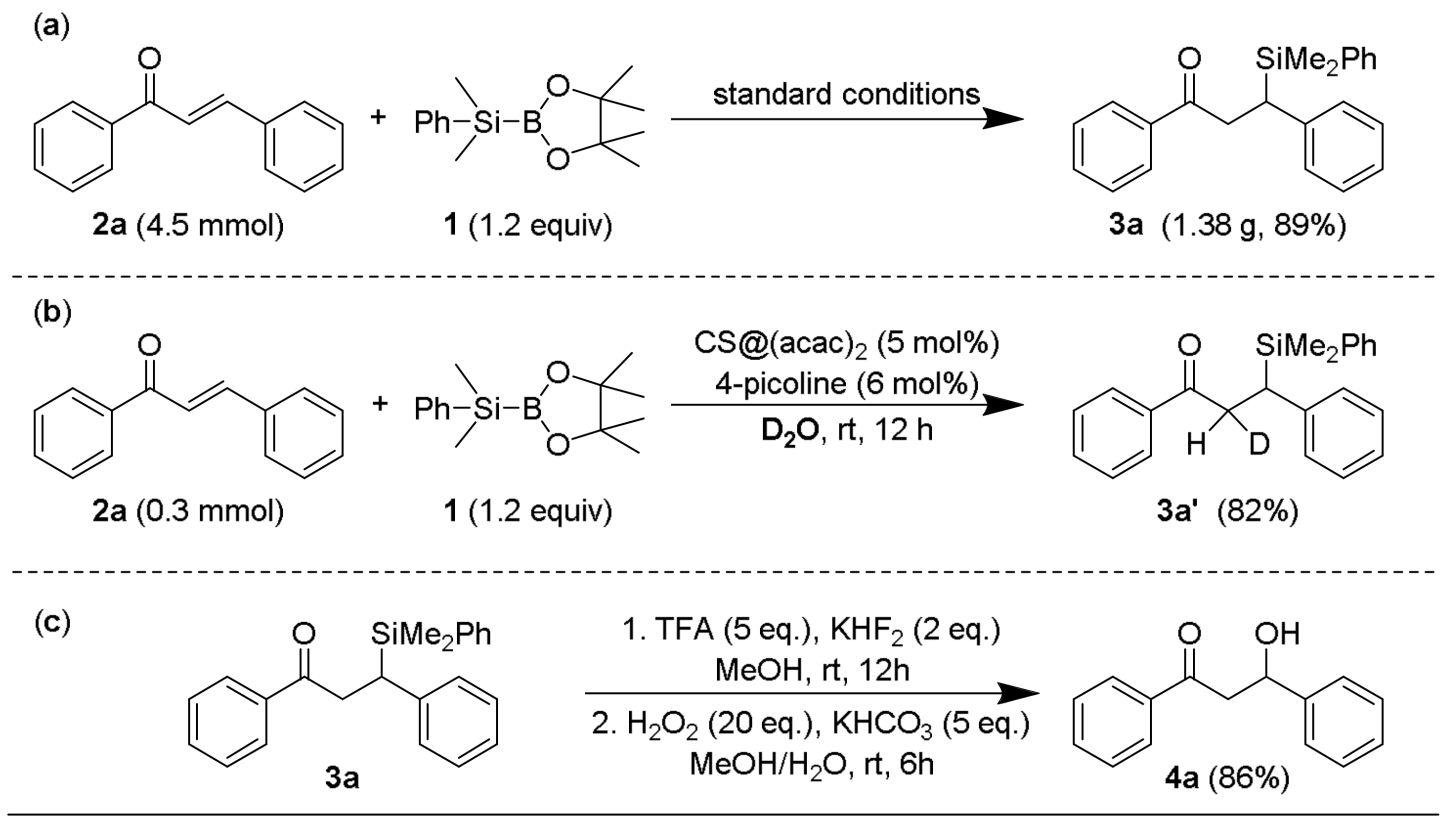
(**a**) Gram scale synthesis β-silyl compound; (**b**) reaction in deuterium oxide; (**c**) further conversion of β-silyl products to β-hydroxyl compounds.

For the purpose of practical application, we further carried out this reaction in a gram scale. As shown in Scheme 1a, β-dimethylphenylsilyl substituted chalcone (**3a**) (in Supplementary Materials) was successfully synthesized in 89% yield under the standard conditions. Next, when this reaction was performed in deuterium oxide instead of water, about 50% deuterium was observed in α-methylene, as revealed by ^1^H NMR spectrum (Scheme 1b). This result indicated that water performs not only as a solvent but also as an important proton source for the protonation step of the whole catalytic cycle. To be mentioned, using water in this silyl conjugate addition made our method green and environmental friendly, because water is non-toxic, cheap, and non-flammable unlike organic solvents. In order to confirm the utility of obtained β-silyl products, further conversion of **3a** by Fleming–Tamao oxidation [45] easily produced corresponding β-hydroxyl compound **4a** (Scheme 1c). This process supplied a simple and efficient protocol to introduce hydroxyl group into complicated structure of natural products and biological active compounds.

**Figure 2:**
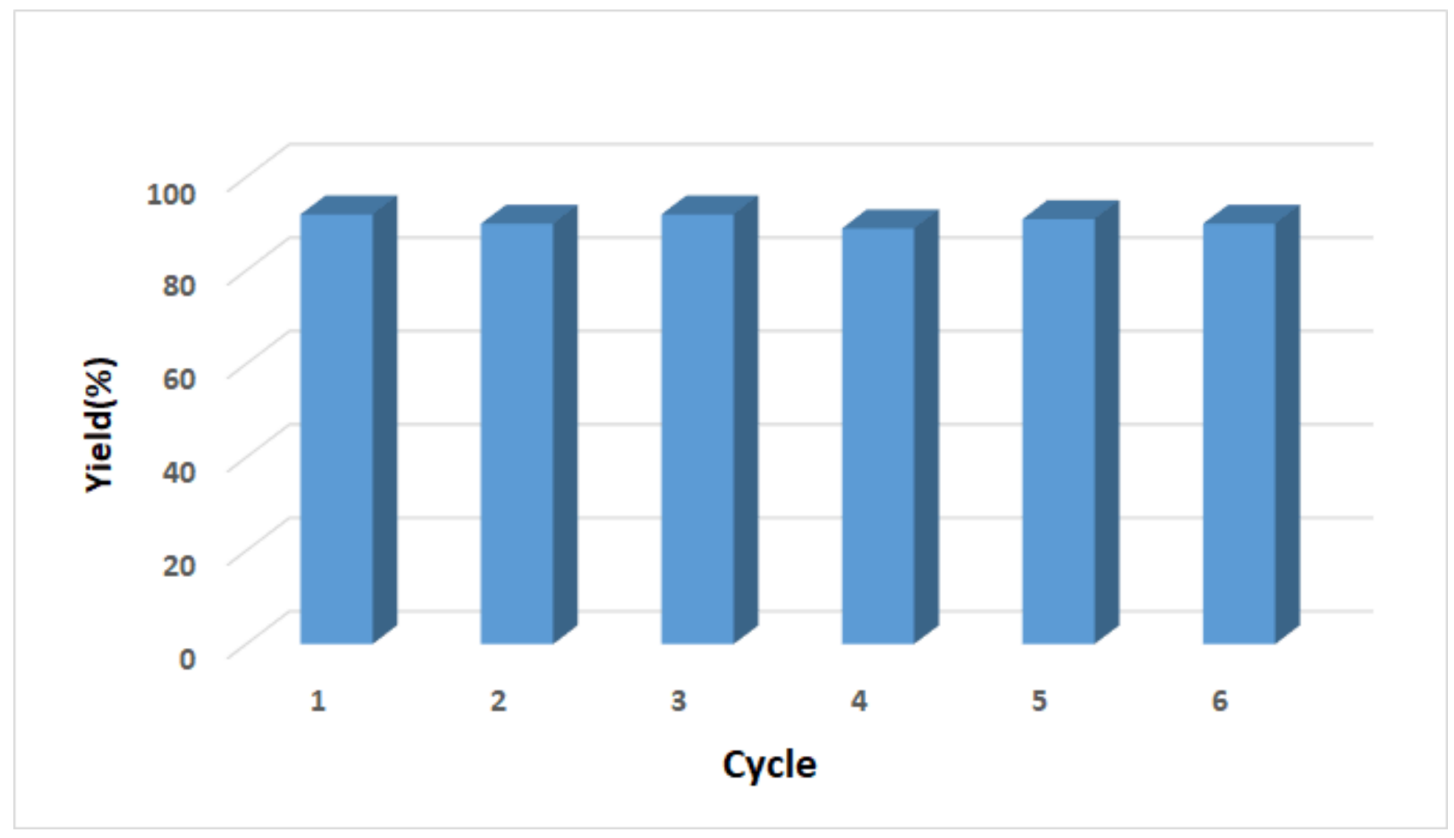
Recycling of chitosan supported copper catalyst.

Recovery and recycling of heterogeneous catalyst are usually crucial considerations for transition metal-catalyzed reactions, from the aspects of economy and sustainability. Thus, the recyclability of chitosan supported copper catalyst was evaluated in the reaction of chalcone (**2a**) with **1** as shown in Figure 2. In each cycle, recovered CS@Cu(acac)_2_ was treated with another new substrate for the next run under the optimized conditions. Remarkably, the catalyst still remained catalytically active after being reused six times. To date, there has only been one report about the recycling of catalyst for just two runs in similar silylation of cyclopentenone, along with apparent decline of reactivity [24]. In addition, we halted the reaction after six hours when about 60% conversion of starting material was received. The catalyst was then removed by filtration at the reaction temperature and the residue reacted for the rest time. It was found that n additional product was formed in the absence of catalyst. Finally, ICP analysis of the filtered aqueous solution after reaction showed no detectable leaching of copper. Both the filtration experiment and metal leaching test strongly suggested chitosan supported copper catalysis is heterogeneous in nature. 

## 4. Conclusions

In summary, we have prepared a heterogeneous chitosan supported copper catalyst which is highly active to catalyze the silyl conjugate addition of α,β-unsaturated acceptors with Suginome’s silylboron reagent. The catalytic reaction was performed in good to excellent yields in water under mild conditions. Various substituted acyclic and cyclic α,β-unsaturated ketones, esters proceeded well under the optimized conditions. Besides, α,β,γ,δ-unsaturated acyclic dienones could also be silylated regioselectively to give corresponding 1,4-addition products. Water possibly plays a predominant role in accelerating the protonation step to achieve high yields. Gram scale synthesis and conversion to β-hydroxy compound made this method more versatile. Remarkably, the catalyst could be easily recovered and recycled six cycles without any significant decrease of reactivity. As a development towards the application of chitosan, our method stands a chance over the previously reported methods in terms of substrate scope, operational simplicity, and recyclability of catalyst.

## Figures and Tables

**Table 1:** Optimization of reaction conditions ^a^.

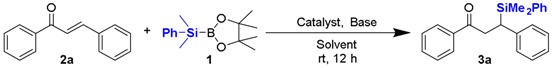				
**Entry**	**Catalyst**	**Base**	**Solvent**	**Yield (%) ^b^**
1	CS@CuSO_4_	none	H_2_O	30
2	CS@Cu(OAc)_2_	none	H_2_O	22
3	CS@Cu(acac)_2_	none	H_2_O	41
4	CS@Cu(acac)_2_	Na_2_CO_3_	H_2_O	50
5	CS@Cu(acac)_2_	K_2_CO_3_	H_2_O	65
6	CS@Cu(acac)_2_	**B1**	H_2_O	40
7	CS@Cu(acac)_2_	**B2**	H_2_O	38
8	CS@Cu(acac)_2_	**B3**	H_2_O	86
9	CS@Cu(acac)_2_	**B4**	H_2_O	92
10	-	**B4**	H_2_O	NR
11	CS@Cu(acac)_2_	**B4**	DCM	NR
12	CS@Cu(acac)_2_	**B4**	THF	NR
13	CS@Cu(acac)_2_	**B4**	Et_2_O	NR
14	CS@Cu(acac)_2_	**B4**	Toluene	NR
15	CS@Cu(acac)_2_	**B4**	MeOH	25
16	CS@Cu(acac)_2_	**B4**	EtOH	8
17 ^c^	CS@Cu(acac)_2_	**B4**	H_2_O	89
18 ^d^	CS@Cu(acac)_2_	**B4**	H_2_O	91
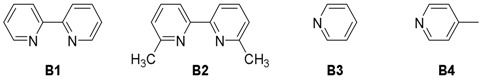				

^a^ Reaction conditions: substrate **2** (0.3 mmol), Me_2_PhSi-B(pin) **1** (1.2 equiv.), catalyst (5 mol % Cu loading), base (6 mol %), H_2_O (2 mL), room temperature, air, 12 h; ^b^ Isolated yield of product; ^c^ 2.5 mol % Cu loading was used; ^d^ Performed under Ar atomosphere.

## Supplementary Materials

The following are available online at http://www.mdpi.com/2073-4360/10/4/385/s1, Characterization data, and spectra for the ^1^H and ^13^C NMR of compounds **3a**–**3q**.
