# Supplementary material for: Recyclable Heterogeneous Chitosan Supported Copper Catalyst for Silyl Conjugate Addition to α,β-Unsaturated Acceptors in Water
Source: Polymers (Basel). 2018 Apr 1;10(4):385. doi: 10.3390/polym10040385 (PMC6415219; doi:10.3390/polym10040385)

**A Recyclable Heterogeneous Chitosan Supported Copper Catalyst for Silyl Conjugate Addition to *α*,*β*-Unsaturated Acceptors in Water**

Lei Zhu,^1,^*, Bojie Li,^1^ Shan Wang,^1^ Wei Wang,^1^ Liansheng Wang,^1^

Liang Ding^2,^* and Caiqin Qin^1,^*

1 School of Chemistry and Materials Science, Hubei Engineering University, Hubei Collaborative Innovation Center of Conversion and Utilization for Biomass Resources, Xiaogan 432000, China

2 Department of Polymer and Composite Material, School of Materials Engineering, Yancheng Institute of Technology, Yancheng 224051, China

**Supporting Information**

**Table of Contents**

| 1. Characterization Data for *β*-Silyl Products | S2-S8 |
| --- | --- |
| 1. ^1^H NMR and ^13^C NMR Spectra | S9-S25 |

1. **Characterization Data for *β*-Silyl Products**

All of adducts are literature-known; obtained characterization data for these compounds is in full agreement with reported data.

3-(dimethyl(phenyl)silyl)-1,3-diphenylpropan-1-one (**3a**)

Colorless oil

^1^H NMR (600 MHz); δ = 0.23 (s, 3H), 0.28 (s, 3H), 3.07 (dd, *J* = 6.6, 10.8 Hz, 1H), 3.18-3.21 (m, 1H), 3.45-3.50 (m, 1H), 6.96 (d, *J* = 8.4 Hz, 2H), 7.05 (t, *J* = 6.6 Hz, 1H), 7.16 (t, *J* = 7.2 Hz, 2H), 7.32-7.39 (m, 5H), 7.42-7.44 (m, 2H), 7.48-7.50 (m, 1H), 7.76 (d, *J* = 7.8 Hz, 2H).

^13^C NMR (150 MHz); δ = -5.3, -3.9, 31.0, 38.9, 124.8, 127.65, 127.72, 127.9, 128.0, 128.4, 129.3, 132.7, 134.1, 136.8, 137.0, 142.3, 199.2.

3-(2-chlorophenyl)-3-(dimethyl(phenyl)silyl)-1-phenylpropan-1-one (**3b**)

Colorless oil.

^1^H NMR (600 MHz); δ = 0.28 (s, 3H), 0.32 (s, 3H), 3.22-3.26 (m, 1H), 3.40-3.45 (m, 1H), 3.73-3.75 (m, 1H), 6.90-6.92 (m, 1H), 6.99-7.01 (m, 1H), 7.07-7.10 (m, 1H), 7.31-7.40 (m, 6H), 7.48-7.51 (m, 3H), 7.74-7.76 (m, 2H).

^13^C NMR (150 MHz); δ = -5.5, -3.6, 26.8, 39.3, 125.7, 126.5, 127.4, 127.8, 127.9, 128.4, 129.4, 129.6, 132.8, 133.8, 134.2, 136.6, 136.8, 140.6, 198.6.

3-(4-chlorophenyl)-3-(dimethyl(phenyl)silyl)-1-phenylpropan-1-one (**3c**)

Colorless oil.

^1^H NMR (600 MHz); δ = 0.25 (s, 3H), 0.29 (s, 3H), 3.04-3.07 (m, 1H), 3.18-3.21 (m, 1H), 3.40-3.45 (m, 1H), 6.87 (d, *J* = 8.4 Hz, 2H), 7.12-7.13 (m, 2H), 7.34-7.43 (m, 7H), 7.50-7.53 (m, 1H), 7.77-7.79 (m, 2H).

^13^C NMR (150 MHz); δ = -5.2, -4.0, 30.7, 38.7, 127.86, 127.91, 128.2, 128.5, 128.9, 129.5, 130.4, 132.9, 134.1, 136.3, 137.0, 141.0, 198.8.

3-(dimethyl(phenyl)silyl)-1-(4-methoxyphenyl)-3-phenylpropan-1-one (**3d**)

Colorless oil.

^1^H NMR (600 MHz); δ = 0.23 (s, 3H), 0.29 (s, 3H), 3.00-3.02 (m, 1H), 3.16-3.19 (m, 1H), 3.39-3.43 (m, 1H), 3.74 (s, 3H), 6.72-6.73 (m, 2H), 6.88-6.89 (m, 2H), 7.33-7.39 (m, 5H), 7.44-7.45 (m, 2H), 7.48-7.50 (m, 1H), 7.77 (d, *J* = 7.2 Hz, 2H).

^13^C NMR (150 MHz); δ =-5.2, -3.8, 30.1, 39.2, 55.1, 113.6, 127.8, 128.0, 128.4, 128.6, 129.3, 132.7, 134.19, 134.21, 137.0, 137.1, 157.0, 199.4.

3-(dimethyl(phenyl)silyl)-1-(4-fluorophenyl)-3-(p-tolyl)propan-1-one (**3e**)

Colorless oil.

^1^H NMR (600 MHz); δ = 0.23 (s, 3H), 0.28 (s, 3H), 2.37 (s, 3H), 3.04-3.06 (m, 1H), 3.15-3.18 (m, 1H), 3.37-3.41 (m, 1H), 6.83-6.90 (m, 4H), 7.18 (d, *J* = 8.4 Hz, 2H), 7.34-7.42 (m, 5H), 7.68 (d, *J* = 7.8 Hz, 2H).

^13^C NMR (150 MHz); δ = -5.1, -4.0, 21.6, 30.5, 38.9, 114.8, 114.9, 127.8, 128.1, 128.8, 128.9, 129.2, 129.4, 134.1, 134.5, 143.6, 198.6.

3-(4-chlorophenyl)-3-(dimethyl(phenyl)silyl)-1-(4-fluorophenyl)propan-1-one (**3f**)

Colorless oil.

^1^H NMR (600 MHz); δ = 0.24 (d, *J* = 9.0 Hz, 3H), 0.28 (d, *J* = 8.4 Hz, 3H), 3.02-3.05 (m, 1H), 3.15-3.19 (m, 1H), 3.37-3.42 (m, 1H), 6.88-6.90 (m, 2H), 7.03-7.06 (m, 2H), 7.13-7.14 (m, 2H), 7.34-7.43 (m, 5H), 7.78-7.81 (m, 2H).

^13^C NMR (150 MHz); δ = -5.2, -3.9, 30.9, 38.8, 115.6, 115.8, 128.0, 128.3, 128.9, 129.4, 129.6, 129.7, 130.5, 130.6, 130.7, 131.2, 133.40, 133.42, 134.2, 136.6, 141.0, 143.4, 164.9, 166.5, 197.3.

4-(Dimethyl(phenyl)silyl)-4-phenylbutan-2-one (**3g**)

Colorless oil.

^1^H NMR (600 MHz); δ = 0.20 (s, 3H), 0.23 (s, 3H), 1.94 (s, 3H), 2.61-2.64 (m, 1H), 2.86-2.93 (m, 2H), 6.92-6.94 (m, 2H), 7.06 (t, *J* = 7.2 Hz, 1H), 7.18 (t, *J* = 7.2 Hz, 2H), 7.33-7.39 (m, 5H).

^13^C NMR (150 MHz); δ = -5.4, -4.1, 29.8, 31.3, 43.8, 124.9, 127.6, 127.7, 128.2, 129.3, 134.1, 136.6, 141.9, 200.6.

3-(Dimethyl(phenyl)silyl)-1-phenylbutan-1-one (**3h**)

Colorless oil.

^1^H NMR (600 MHz); δ = 0.35 (d, *J* = 1.2 Hz, 3H), 0.35 (d, *J* = 0.6 Hz, 3H), 0.99-1.01 (m, 3H), 1.62-1.67 (m, 1H), 2.65-2.69 (m, 1H), 3.00-3.03 (m, 1H), 7.38-7.43 (m, 5H), 7.52-7.57 (m, 3H), 7.83-7.85 (m, 2H).

^13^C NMR (150 MHz); δ = -5.4, -4.8, 14.5, 15.9, 40.6, 127.8, 128.1, 128.5, 129.1, 132.7, 133.9, 137.1, 137.6, 200.6.

3-(dimethyl(phenyl)silyl)-4,4-dimethyl-1-phenylpentan-1-one (**3i**)

Colorless oil.

^1^H NMR (600 MHz); δ = 0.32 (s, 3H), 0.39 (s, 3H), 0.90 (s, 9H), 2.13 (t, *J* = 5.4 Hz, 1H), 2.94-2.98 (m, 1H), 3.04-3.08 (m, 1H), 7.26-7.28 (m, 3H), 7.41 (t, *J* = 7.8 Hz, 2H), 7.50-7.55 (m, 3H), 7.84-7.85 (m, 2H).

^13^C NMR (150 MHz); δ = -1.4, -0.4, 30.5, 31.3, 34.2, 37.0, 127.7, 127.8, 128.4, 128.7, 132.6, 134.0, 137.3, 140.0, 200.0.

Methyl-3-(dimethyl(phenyl)silyl)-3-phenylpropanoate (**3j**)

Yellowish oil.

^1^H NMR (600 MHz); δ = 0.21 (s, 3H), 0.25 (s, 3H), 2.62-2.66 (m, 1H), 2.73-2.78 (m, 1H), 2.83-2.86 (m, 1H), 3.46 (s, 3H), 6.93-6.95 (m, 2H), 7.09 (t, *J* = 7.8 Hz, 1H), 7.18-7.21 (m, 2H), 7.32-7.35 (m, 2H), 7.36-7.41 (m, 3H).

^13^C NMR (150 MHz); δ = -5.5, -4.1, 32.2, 34.7, 51.5, 125.0, 127.5, 127.7, 128.1, 129.3, 134.1, 136.5, 141.7, 173.4.

Ethyl-3-(dimethyl(phenyl)silyl)-3-phenylpropanoate (**3k**)

Yellowish oil.

^1^H NMR (600 MHz); δ = 0.21 (d, *J* = 1.2 Hz, 3H), 0.23 (d, *J* = 1.8 Hz, 3H), 1.02-1.04 (m, 3H), 2.60-2.64 (m, 1H), 2.71-2.76 (m, 1H), 2.82-2.85 (m, 1H), 3.89 (q, *J* = 6.6 Hz, 2H), 6.93 (d, *J* = 8.4 Hz, 2H), 7.09 (t, *J* = 7.2 Hz, 1H), 7.17-7.20 (m, 2H), 7.32-7.40 (m, 5H).

^13^C NMR (150 MHz); δ = -5.5, -4.1, 14.0, 32.3, 34.9, 60.2, 124.9, 127.6, 127.7, 128.0, 129.3, 134.1, 136.5, 173.0.

3-(dimethyl(phenyl)silyl)cyclopentan-1-one (**3l**)

Yellowish oil.

^1^H NMR (600 MHz); δ = 0.33 (d, *J* = 1.2 Hz, 6H), 1.54-1.56 (m, 1H), 1.66-1.70 (m, 1H), 1.87-1.92 (m, 1H), 2.09-2.13 (m, 2H), 2.21-2.30 (m, 2H), 7.36-7.39 (m, 3H), 7.49-7.51 (m, 2H).

^13^C NMR (150 MHz); δ = -5.02, -4.97, 23.9, 24.9, 39.3, 40.1, 127.9, 129.3, 133.8, 136.9, 221.0.

3-(Dimethyl(phenyl)silyl)cyclohexan-1-one (**3m**)

Yellowish oil.

^1^H NMR (600 MHz); δ = 0.30 (d, *J* = 1.8 Hz, 6H), 1.26-1.31 (m, 1H), 1.40-1.43 (m, 1H), 1.66-1.72 (m, 1H), 1.80 (d, *J* = 10.8 Hz, 1H), 2.07-2.15 (m, 2H), 2.26-2.31 (m, 2H), 2.35 (d, *J* = 15.0 Hz, 1H), 7.36-7.39 (m, 3H), 7.47-7.48 (m, 2H).

^13^C NMR (150 MHz); δ = -5.3, -5.2, 26.1, 27.7, 29.8, 42.0, 42.5, 128.0, 129.4, 134.0, 136.7, 212.9.

4-(dimethyl(phenyl)silyl)dihydrofuran-2(3H)-one (**3n**)

Colorless oil

^1^H NMR (600 MHz); δ = 0.36 (t, *J* = 2.4 Hz, 6H), 2.04-2.09 (m, 1H), 2.27-2.31 (m, 1H), 2.48-2.53 (m, 1H), 4.09-4.12 (m, 1H), 4.42 (t, *J* = 8.4 Hz, 1H), 7.38-7.42 (m, 3H), 7.46-7.47 (m, 2H).

^13^C NMR (150 MHz); δ = -5.1, -4.9, 23.8, 30.3, 70.8, 128.3, 129.9, 133.6, 135.1, 178.0.

4-(dimethyl(phenyl)silyl)tetrahydro-2H-pyran-2-one (**3o**)

Colorless oil

^1^H NMR (600 MHz); δ = 0.34 (d, *J* = 1.2 Hz, 6H), 1.39-1.41 (m, 1H), 1.65-1.67 (m, 1H), 1.83-1.84 (m, 1H), 2.25-2.30 (m, 1H), 2.55-2.59 (m, 1H), 4.24-4.27 (m, 1H), 4.32-4.34 (m, 1H), 7.36-7.41 (m, 3H), 7.47-7.49 (m, 2H)..

^13^C NMR (150 MHz); δ = -5.6, -5.5, 18.5, 23.8, 31.0, 70.3, 128.2, 129.8, 133.9, 135.6, 171.7.

*E*-4-(dimethyl(phenyl)silyl)-6-phenylhex-5-en-2-one (**3q**)

Colorless oil

^1^H NMR (600 MHz); δ = 0.33 (s, 6H), 2.05 (s, 3H), 2.44-2.50 (m, 2H), 2.55-2.59 (m, 1H), 6.03-6.08 (m, 1H), 6.18 (d, *J* = 15.6 Hz, 1H), 7.15-7.17 (m, 1H), 7.24-7.26 (m, 5H), 7.36-7.39 (m, 3H), 7.48-7.49 (m, 2H).

^13^C NMR (150 MHz); δ = -5.2, -4.4, 29.1, 29.8, 43.3, 125.7, 126.6, 127.9, 128.3, 128.4, 129.4, 130.4, 134.0, 136.5, 137.8, 208.5.

*E*-3-(dimethyl(phenyl)silyl)-1,5-diphenylpent-4-en-1-one (**3q**)

Colorless oil

^1^H NMR (600 MHz); δ = 0.37 (d, *J* = 1.8 Hz, 3H), 0.40 (d, *J* = 1.8 Hz, 3H), 2.66-2.67 (m, 1H), 3.05-3.10 (m, 2H), 6.12-6.19 (m, 2H), 7.13-7.15 (m, 1H), 7.21-7.25 (m, 4H), 7.36-7.42 (m, 5H), 7.50-7.55 (m, 3H), 7.81-7.80 (m, 2H).

^13^C NMR (150 MHz); δ = -5.0, -4.1, 29.2, 38.2, 125.9, 126.6, 128.0, 128.1, 128.4, 128.6, 129.5, 130.8, 132.9, 134.2, 136.9, 137.1, 138.1, 199.6.

1. **^1^H and ^13^C NMR spectra**

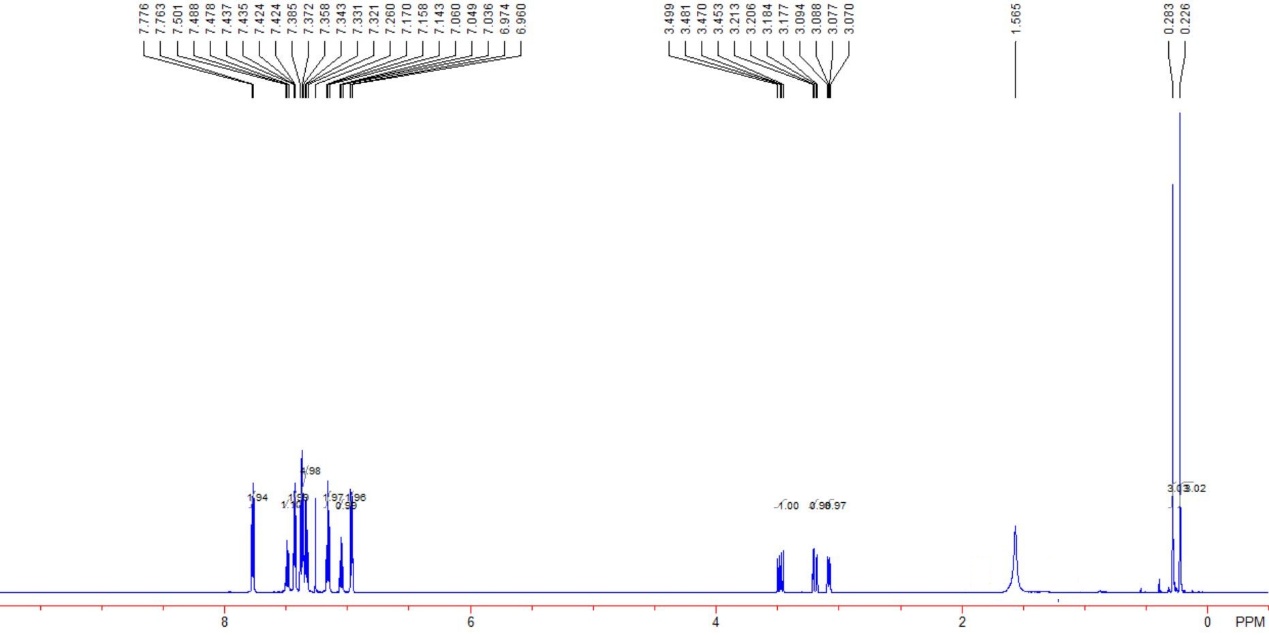


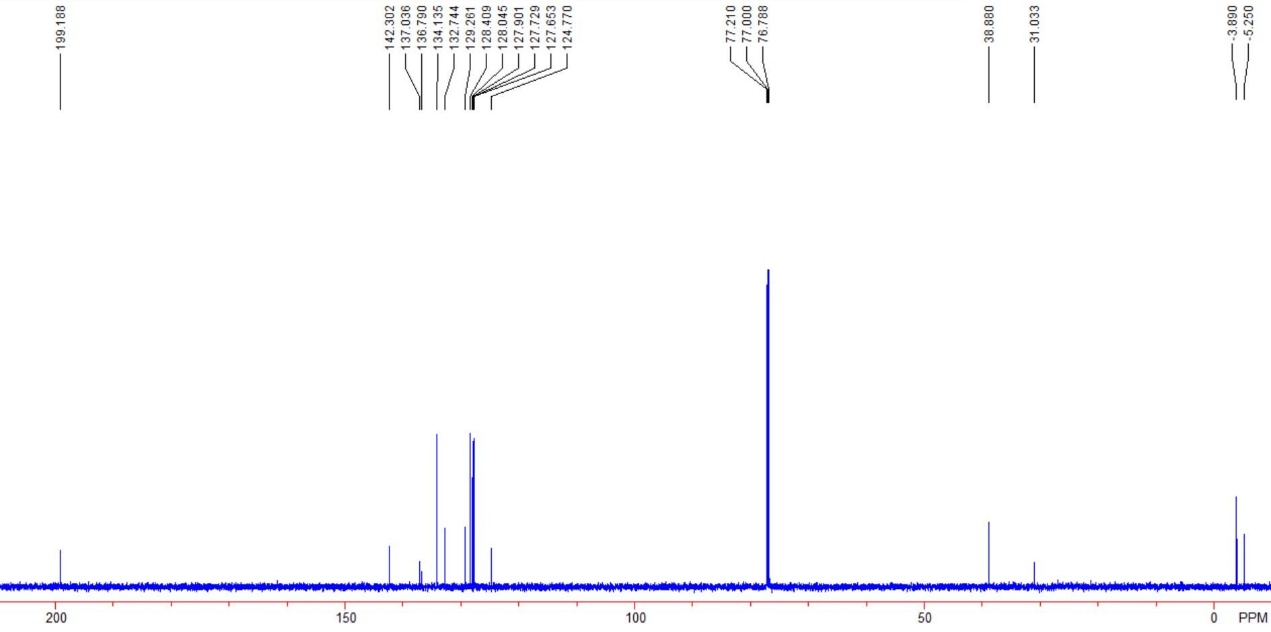

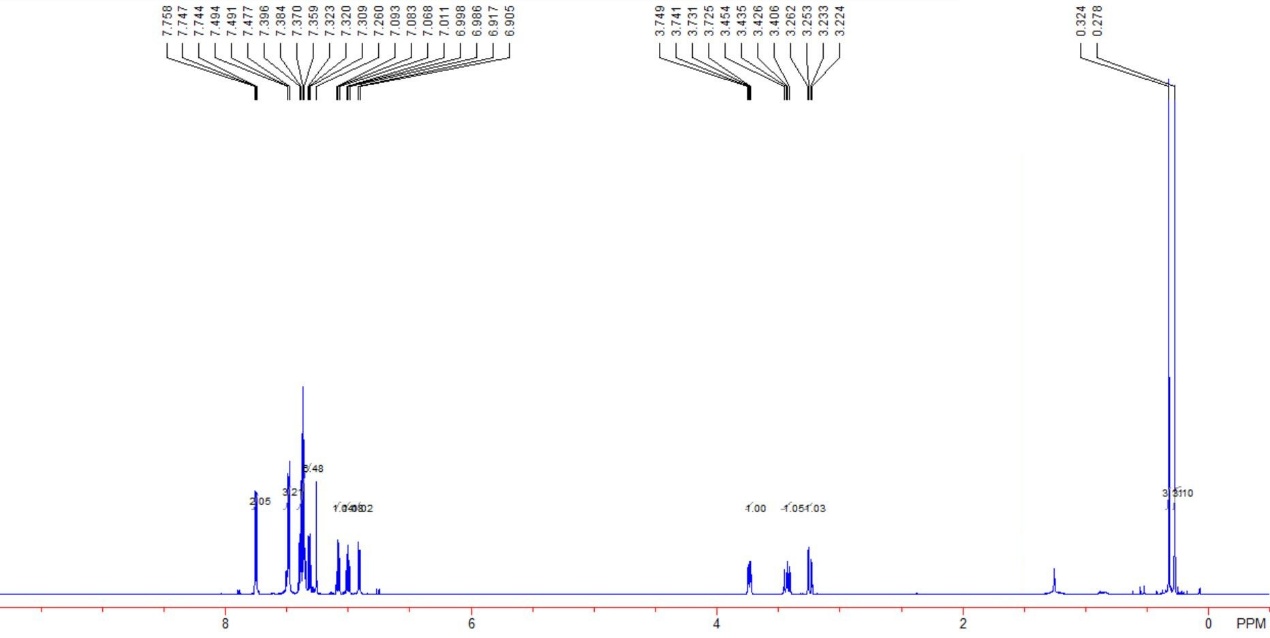


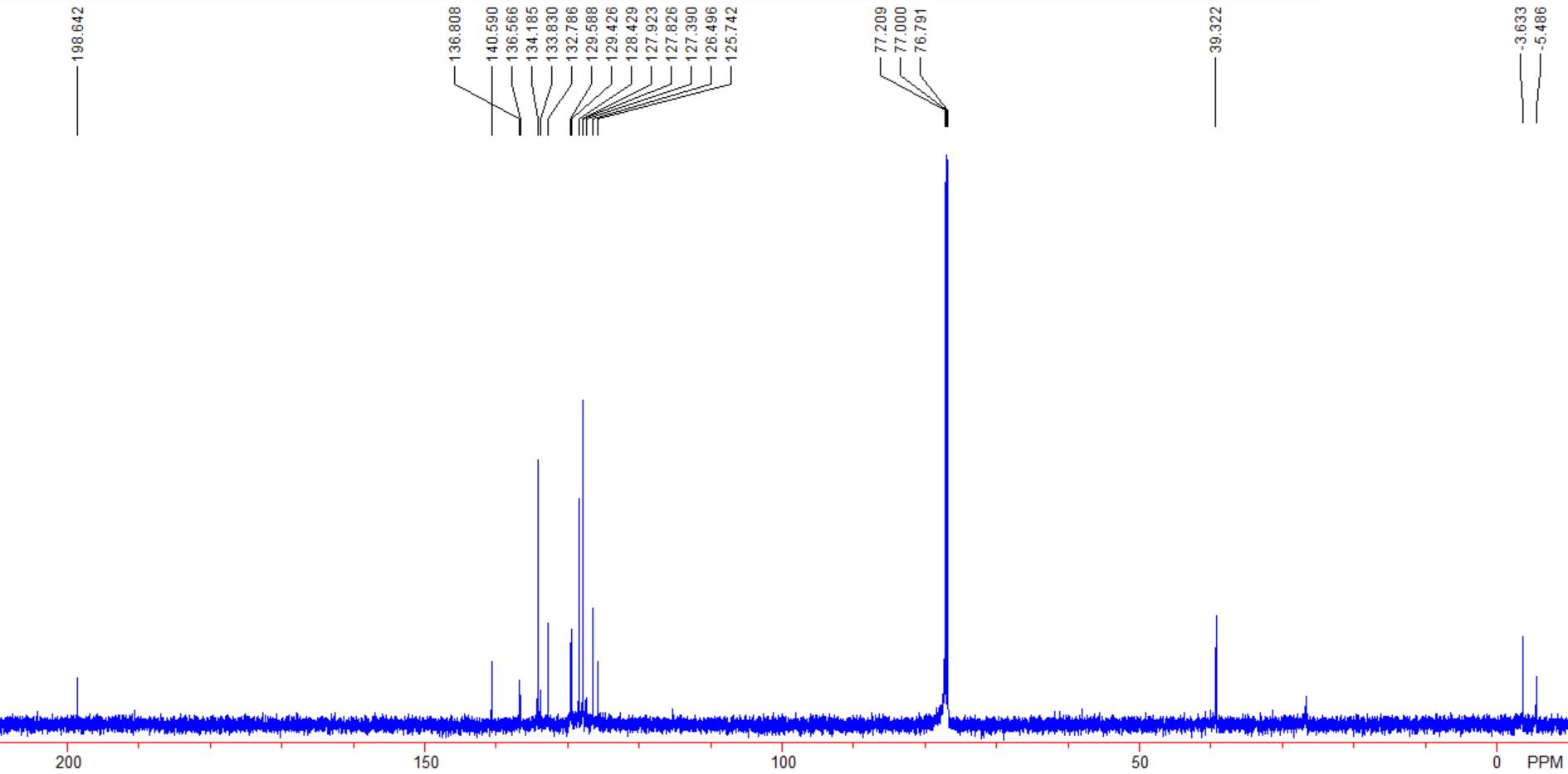

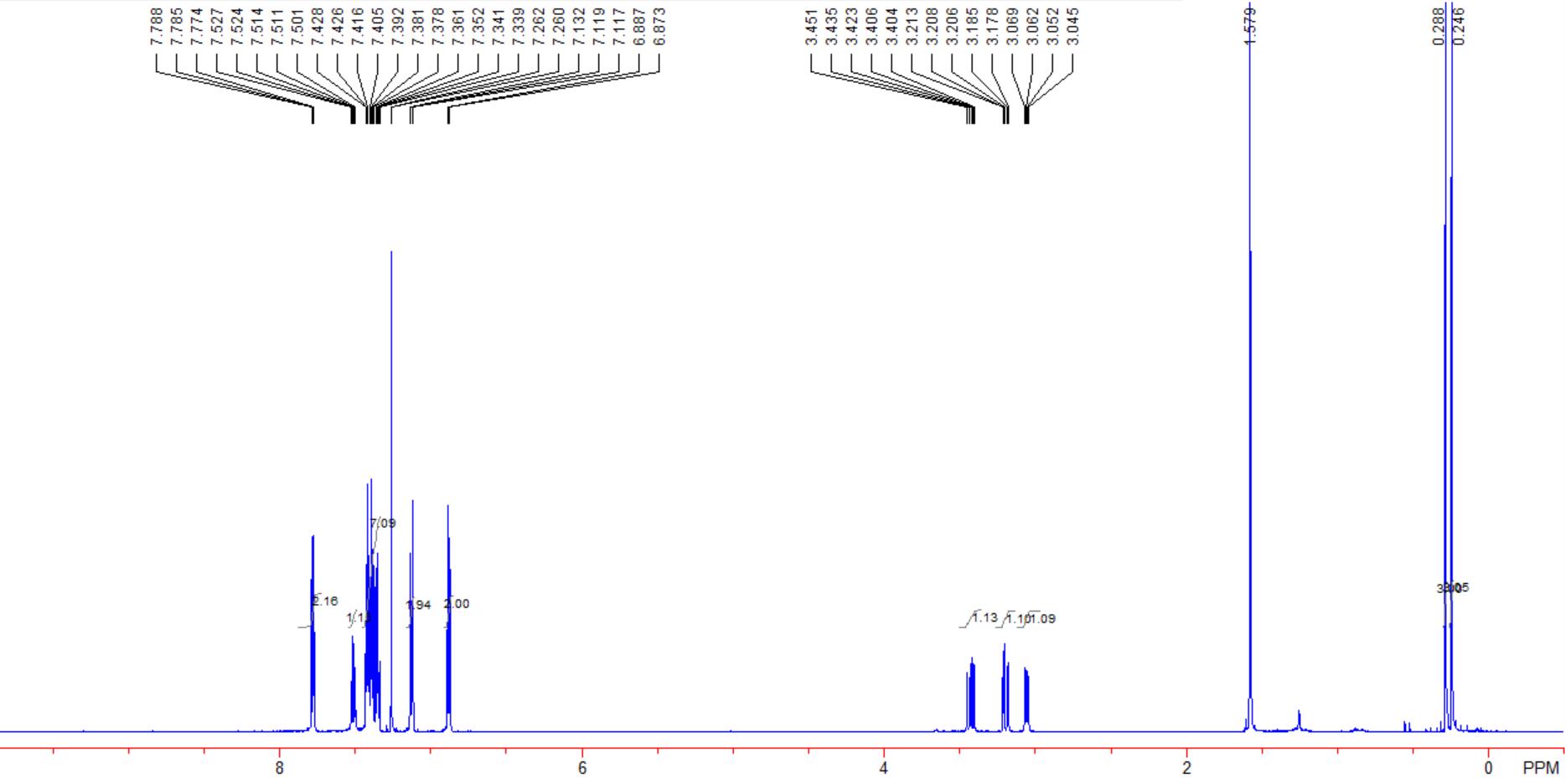


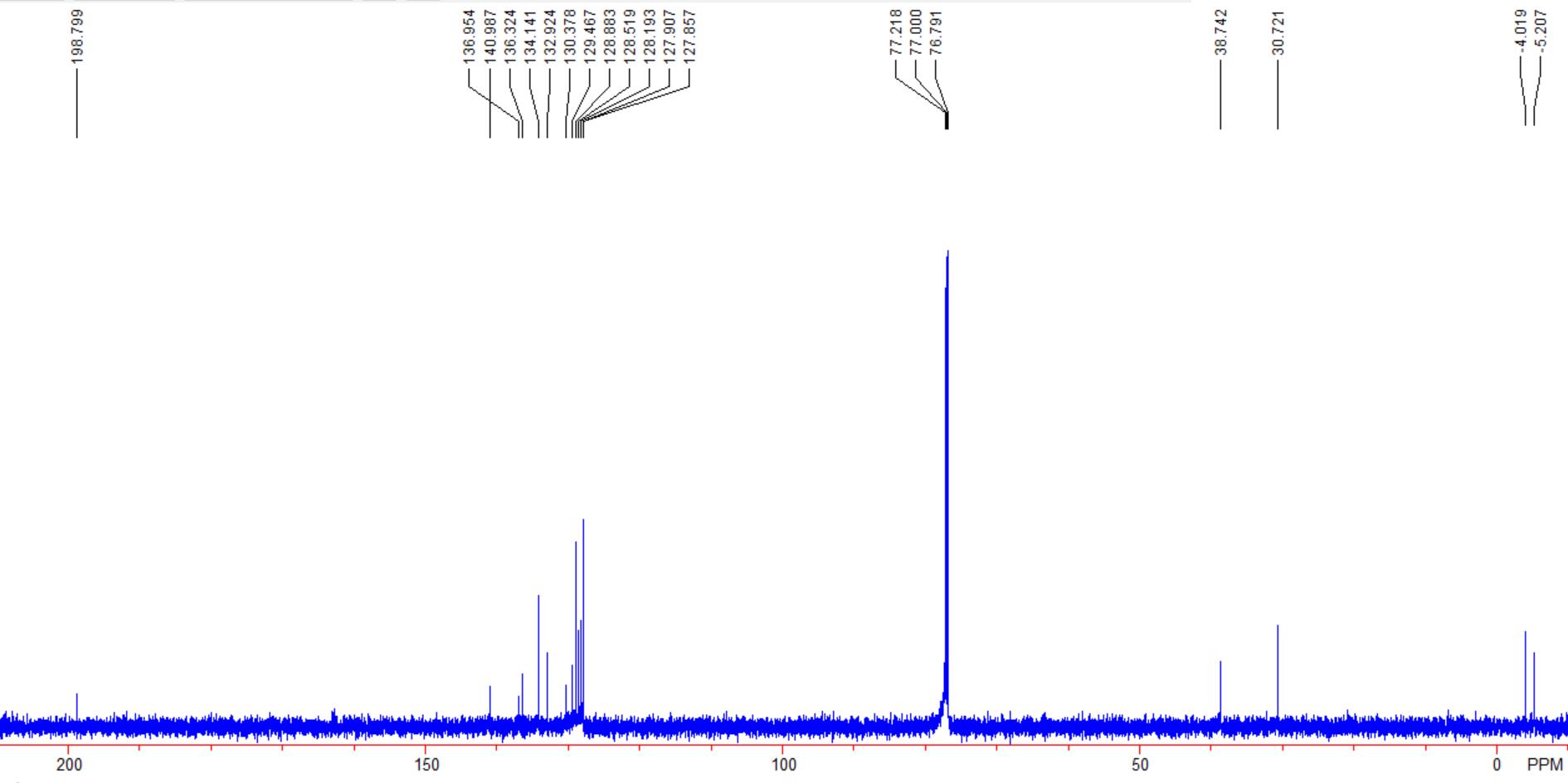

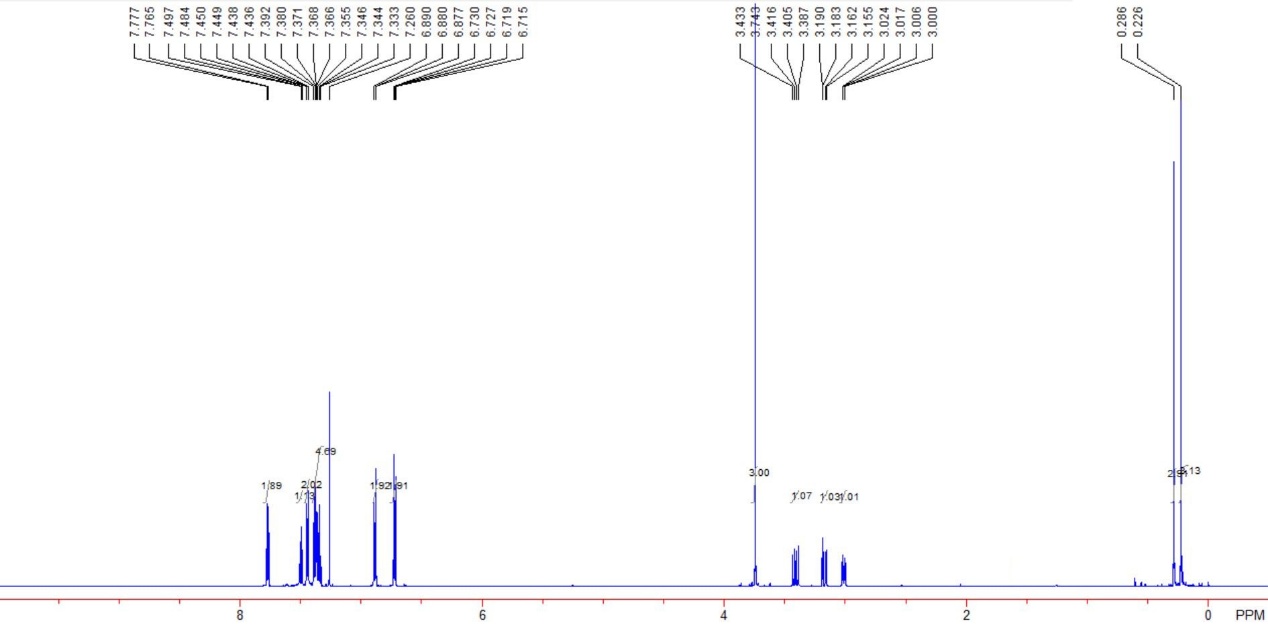


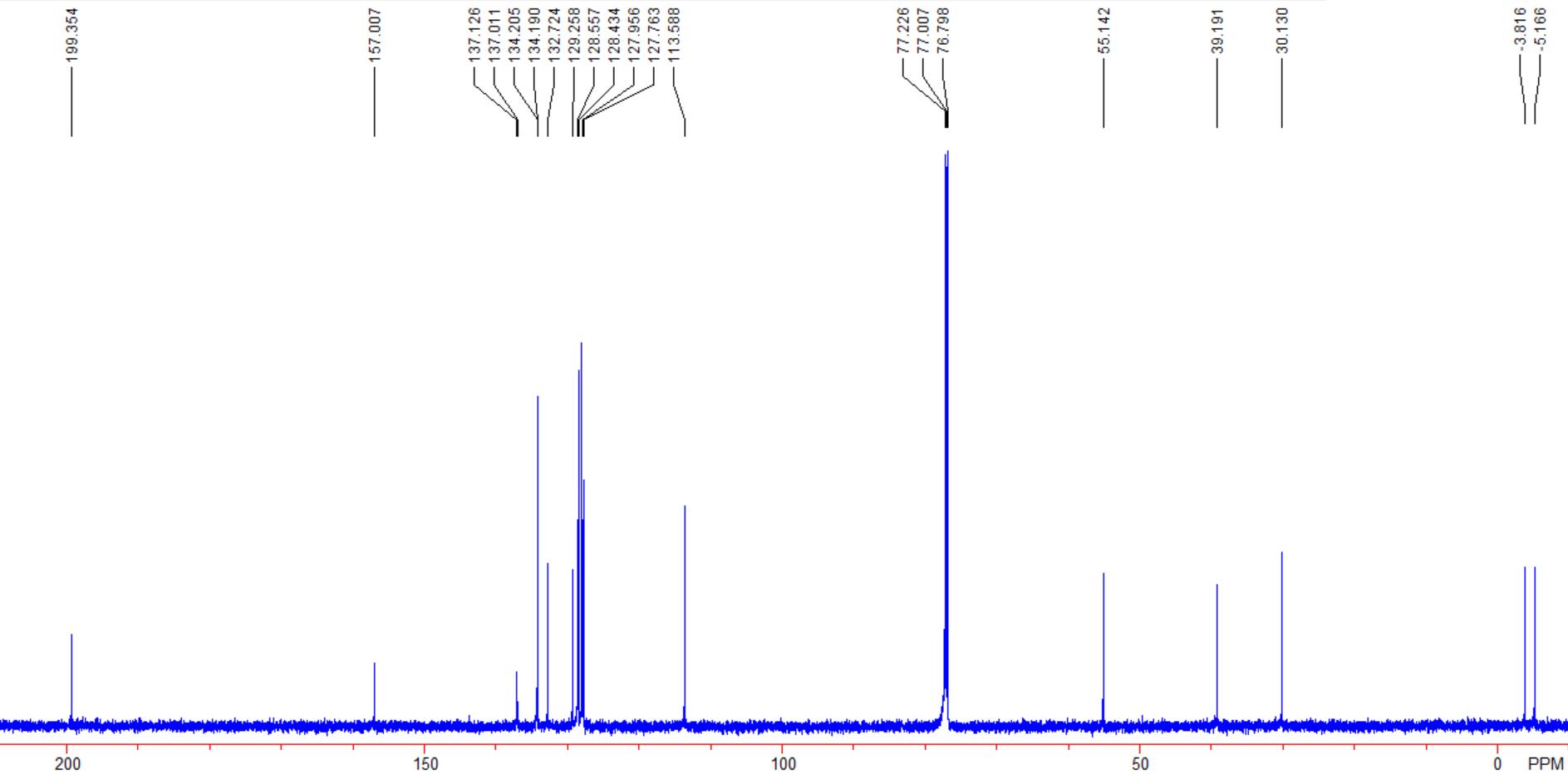

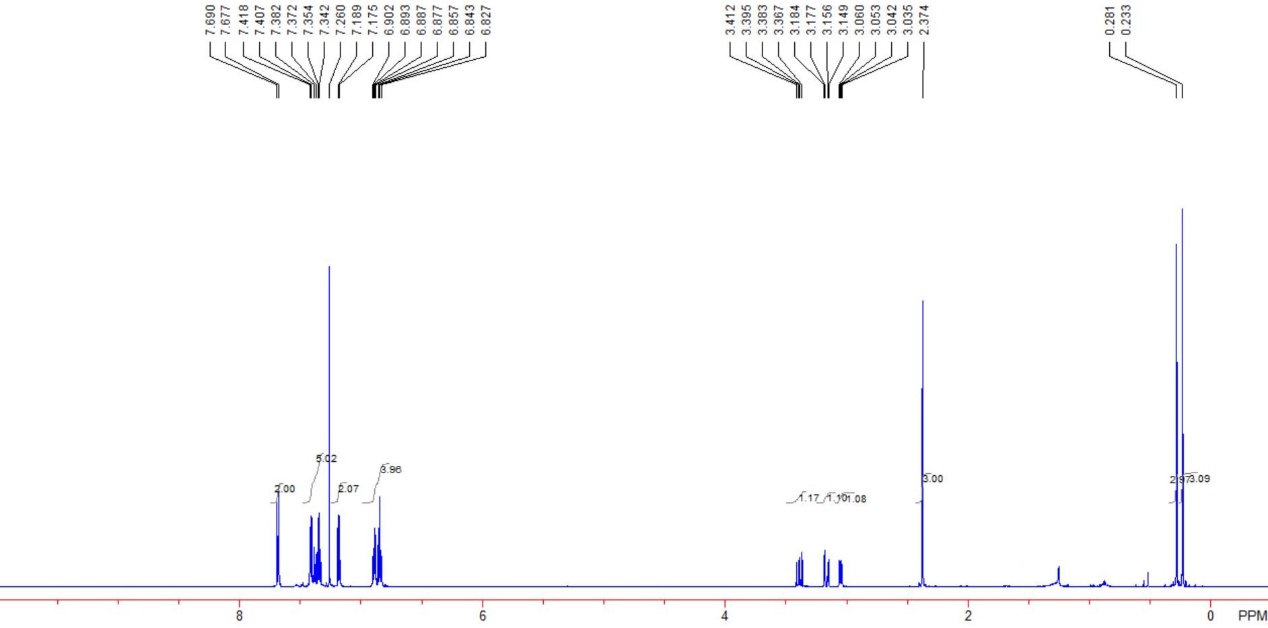


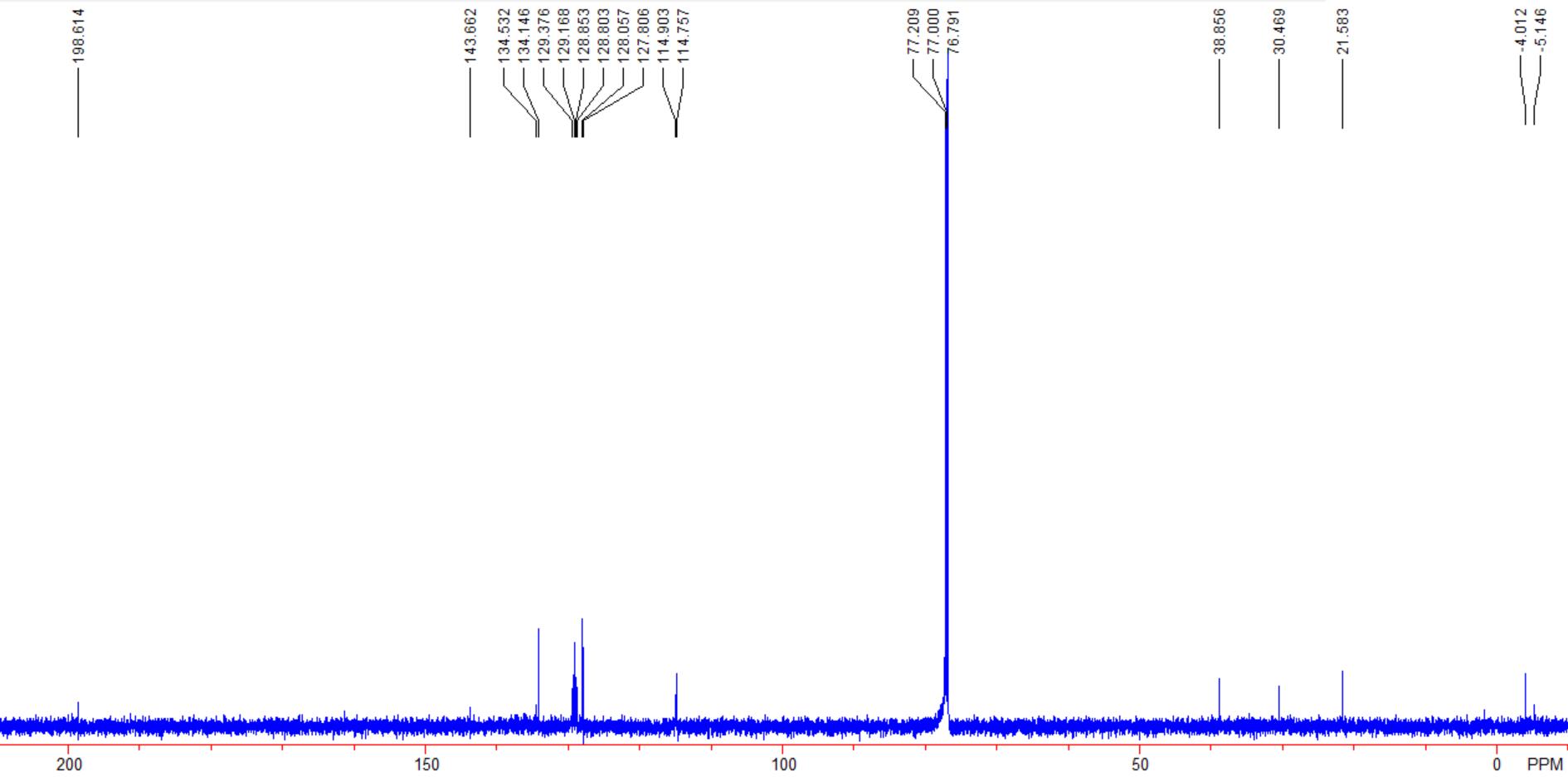

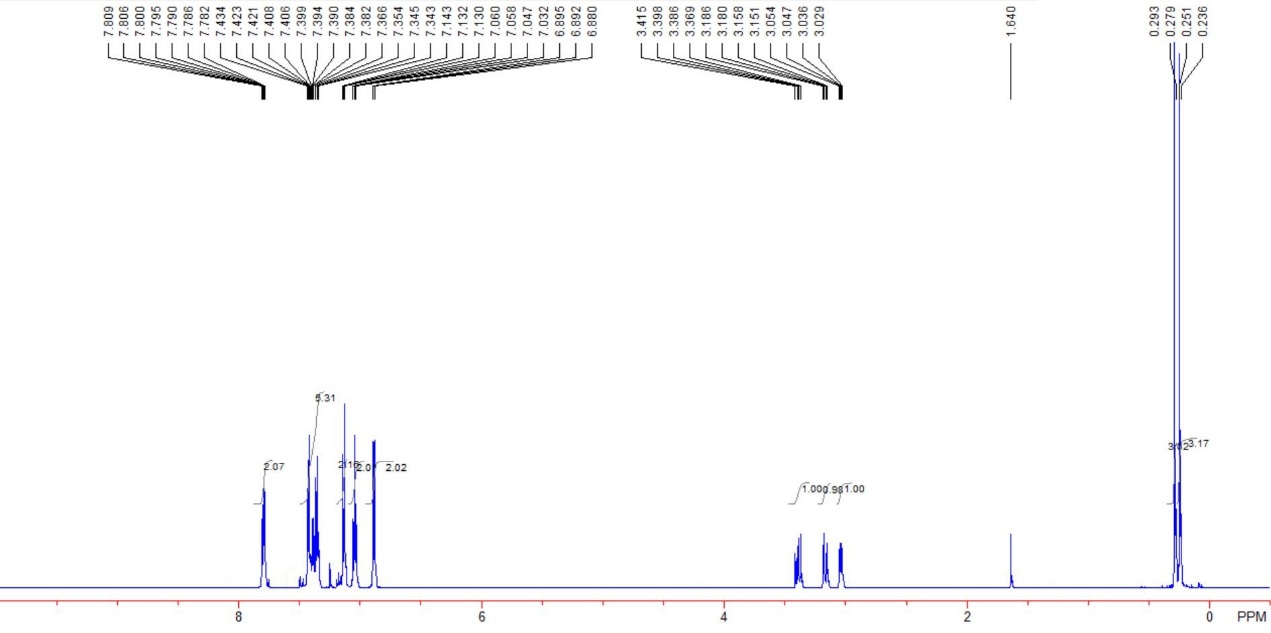


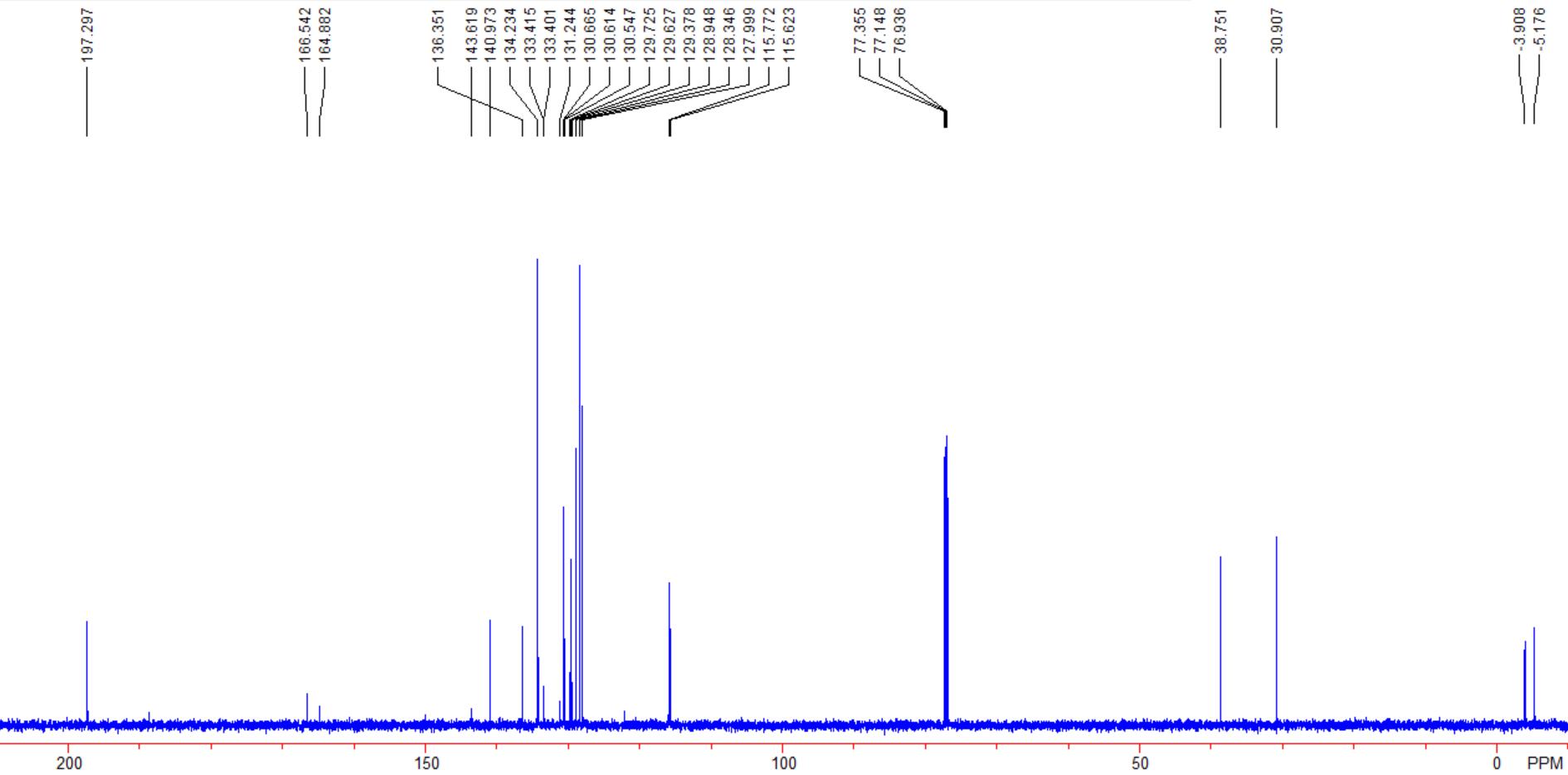

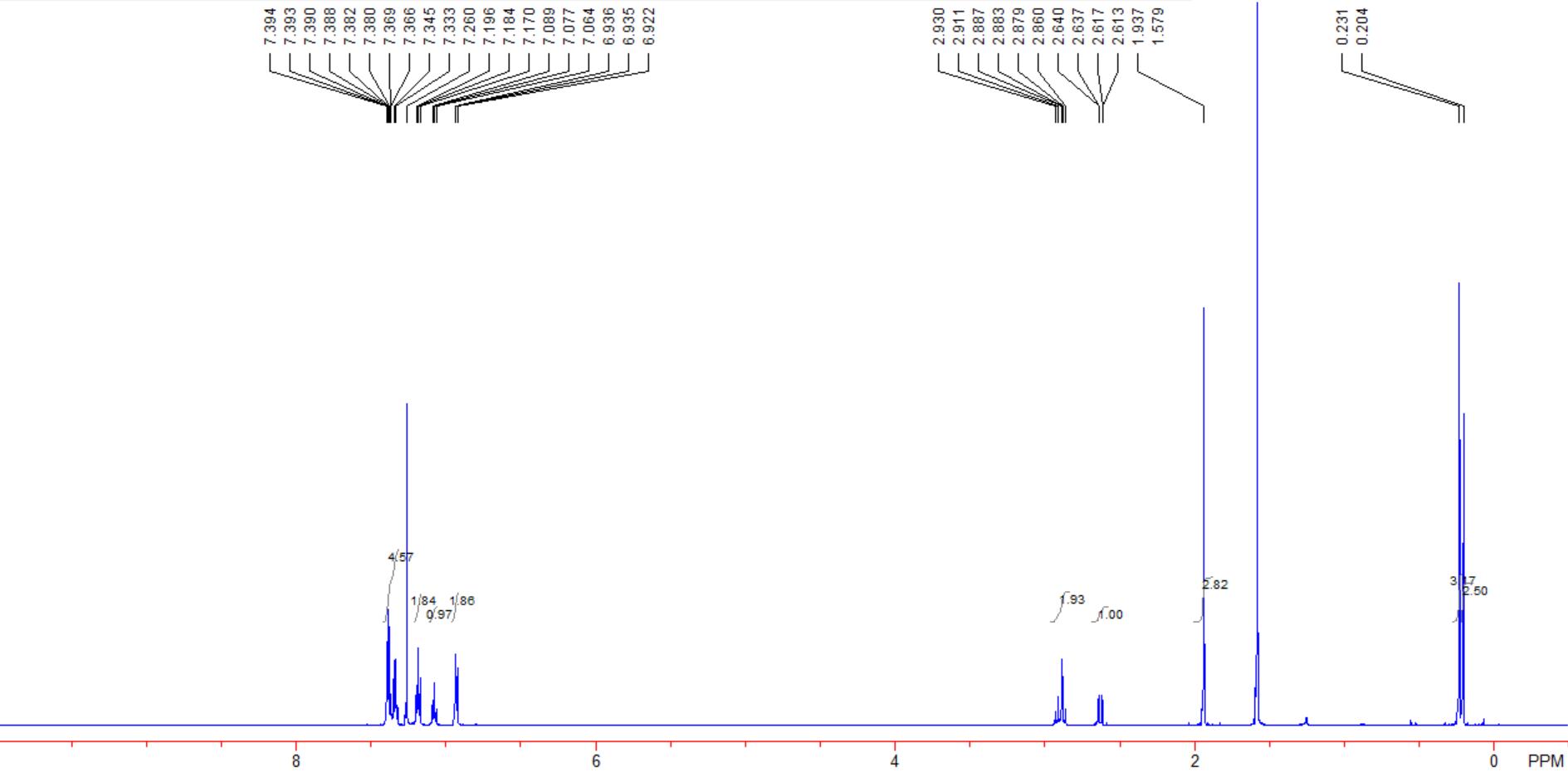


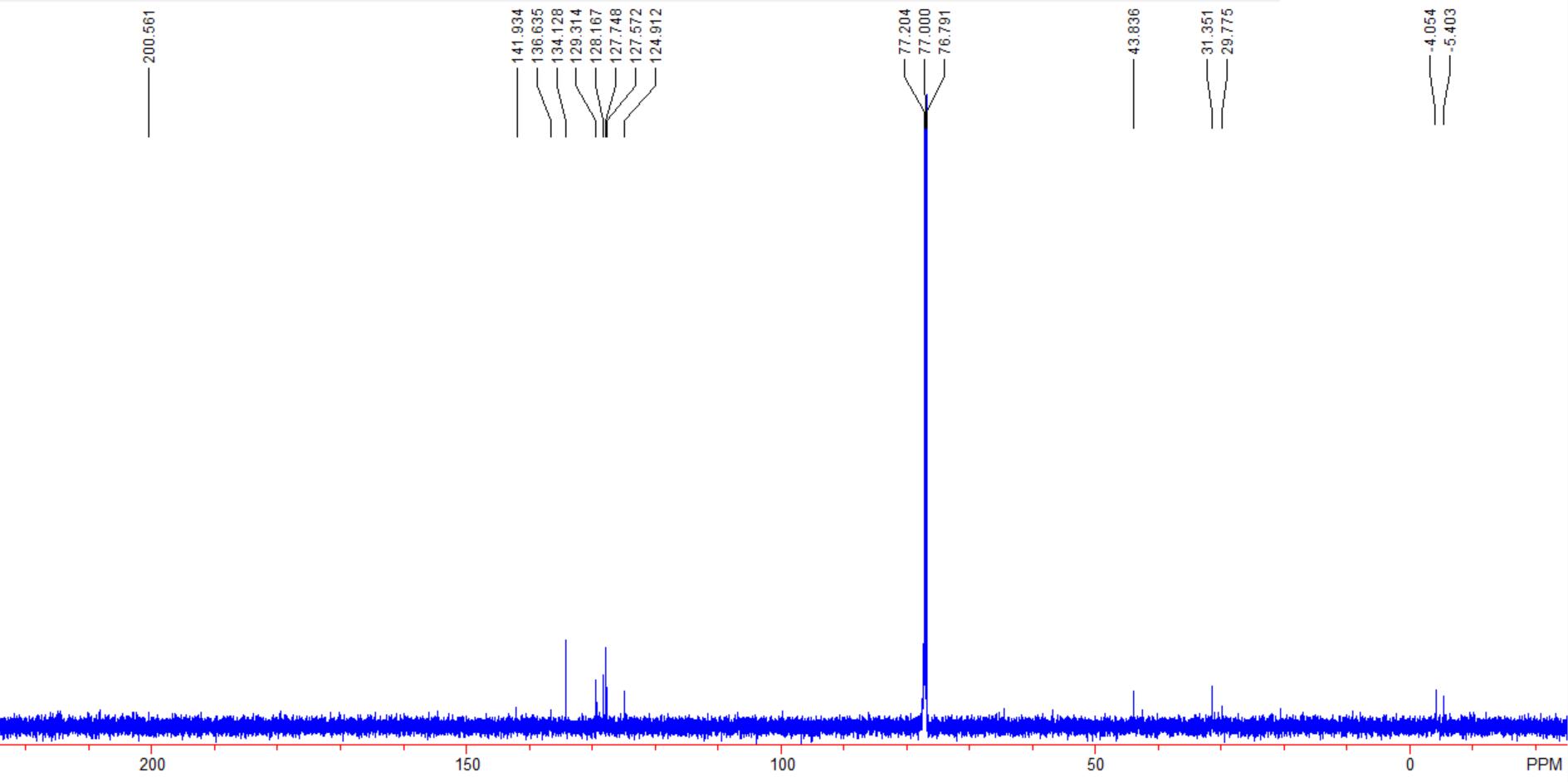

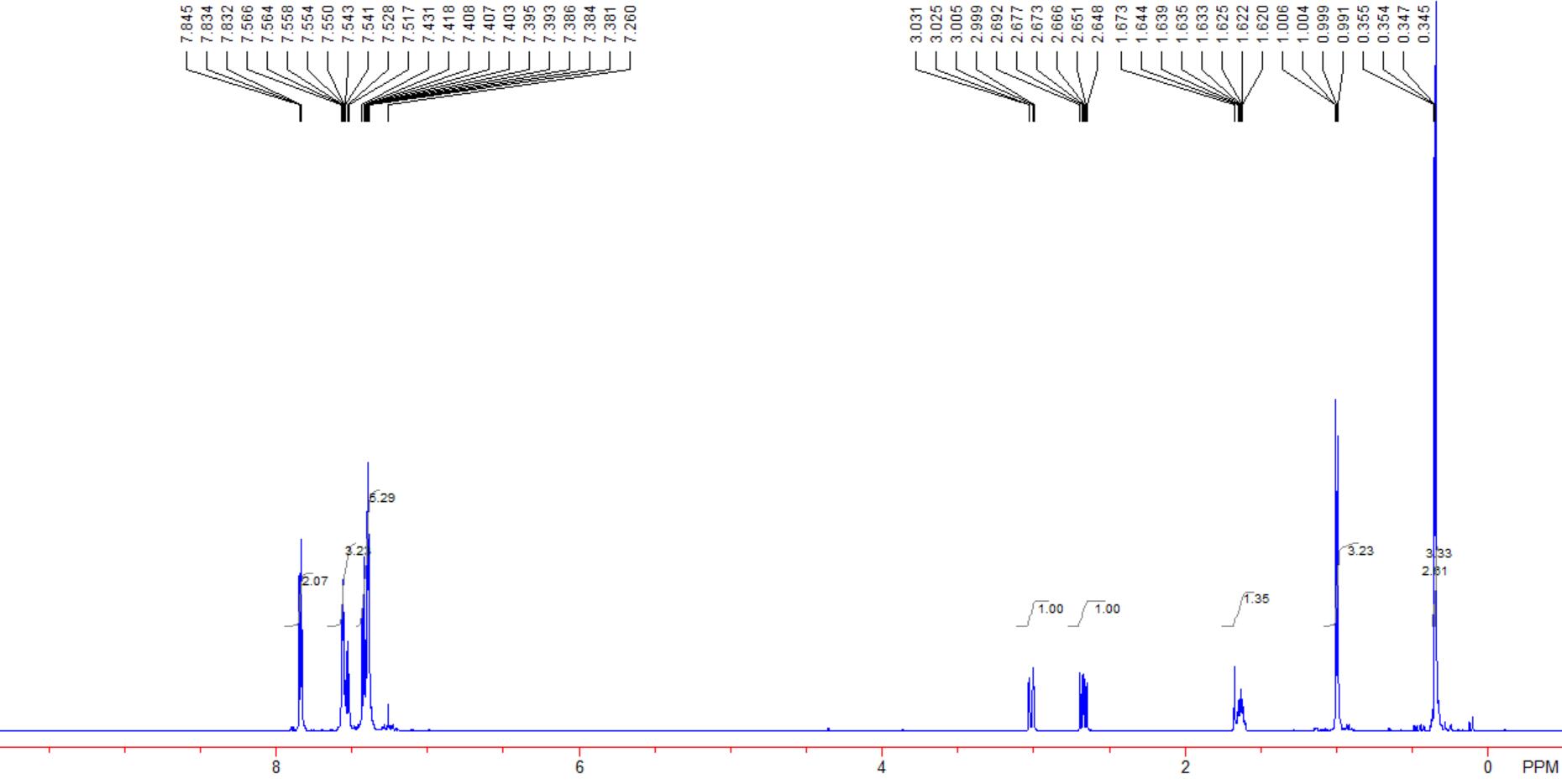


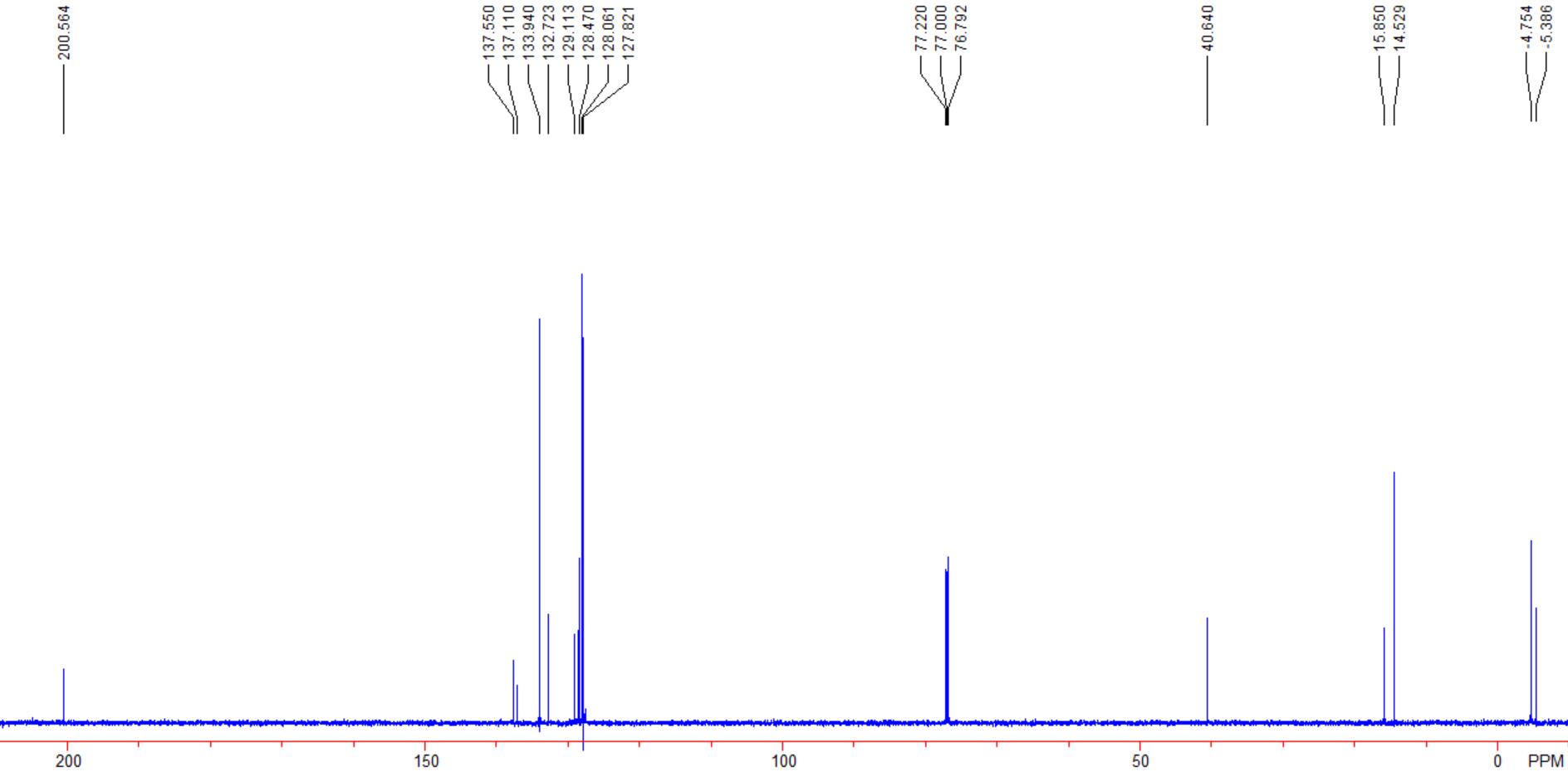

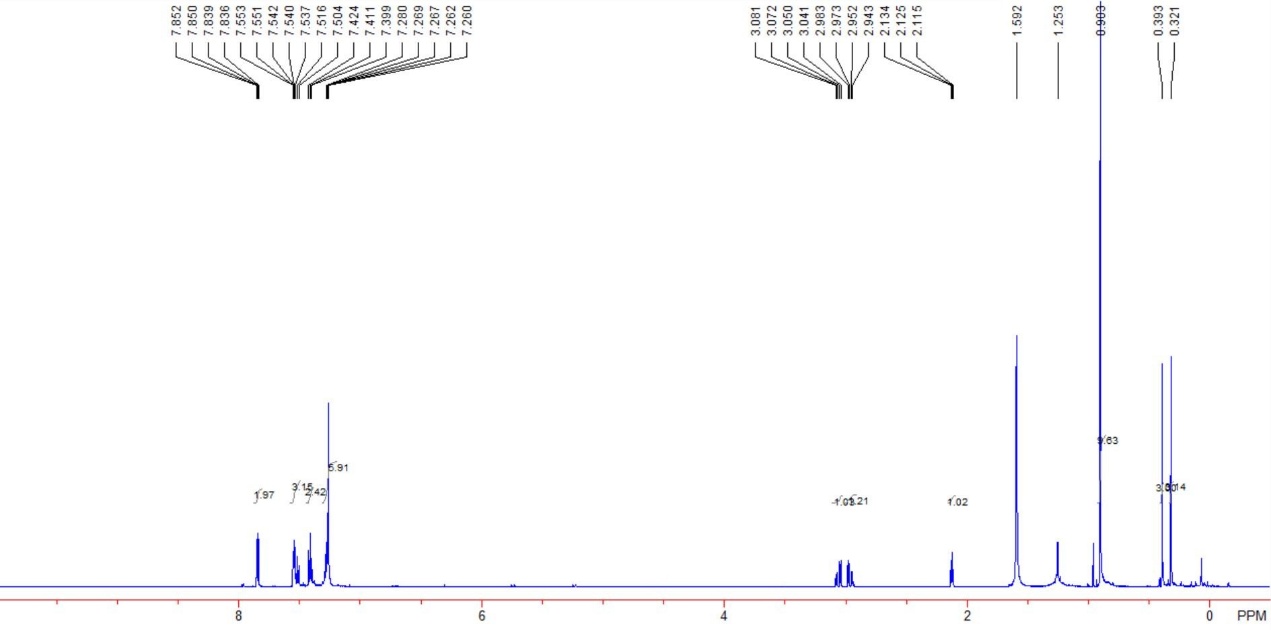


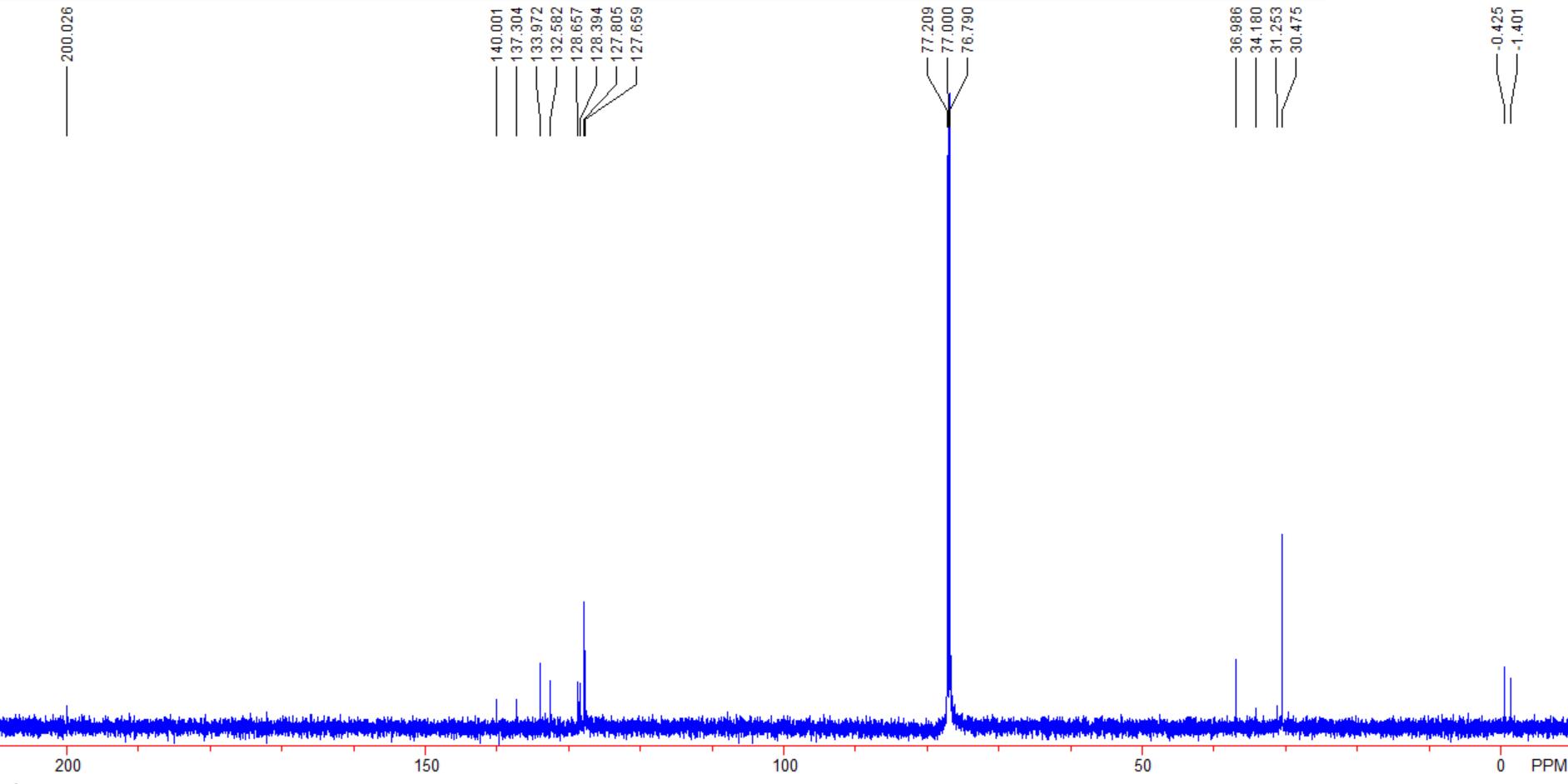

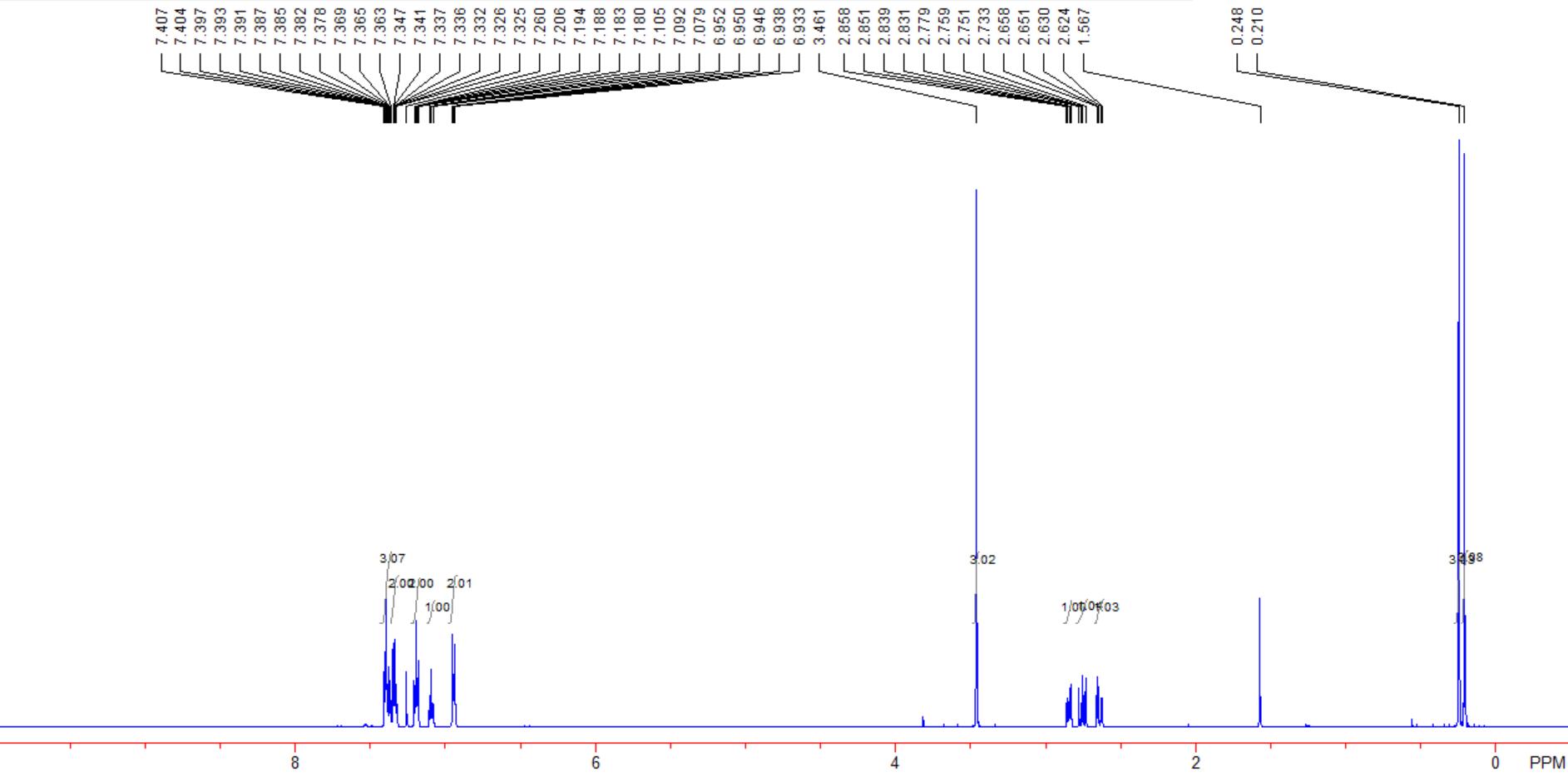


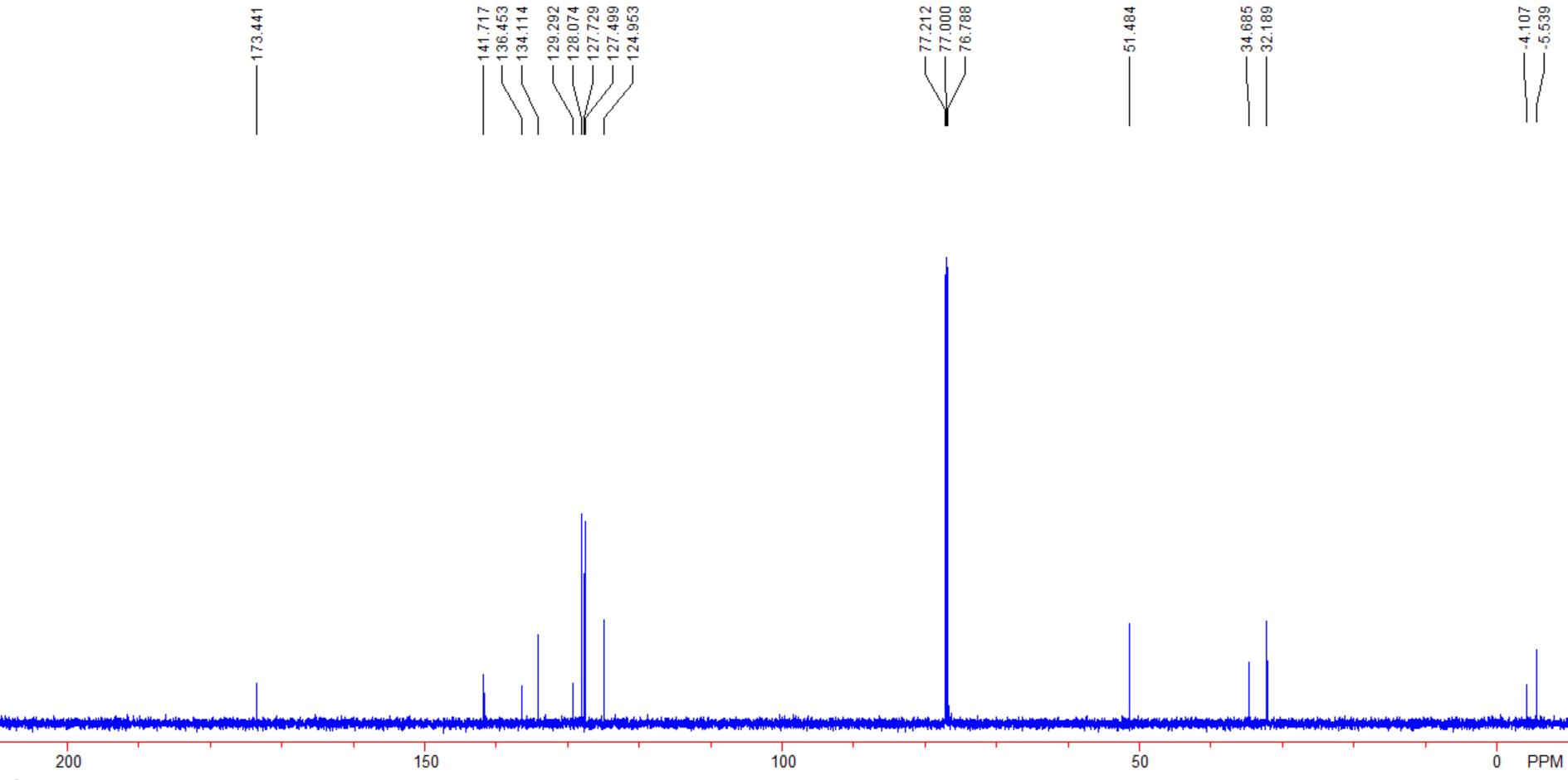

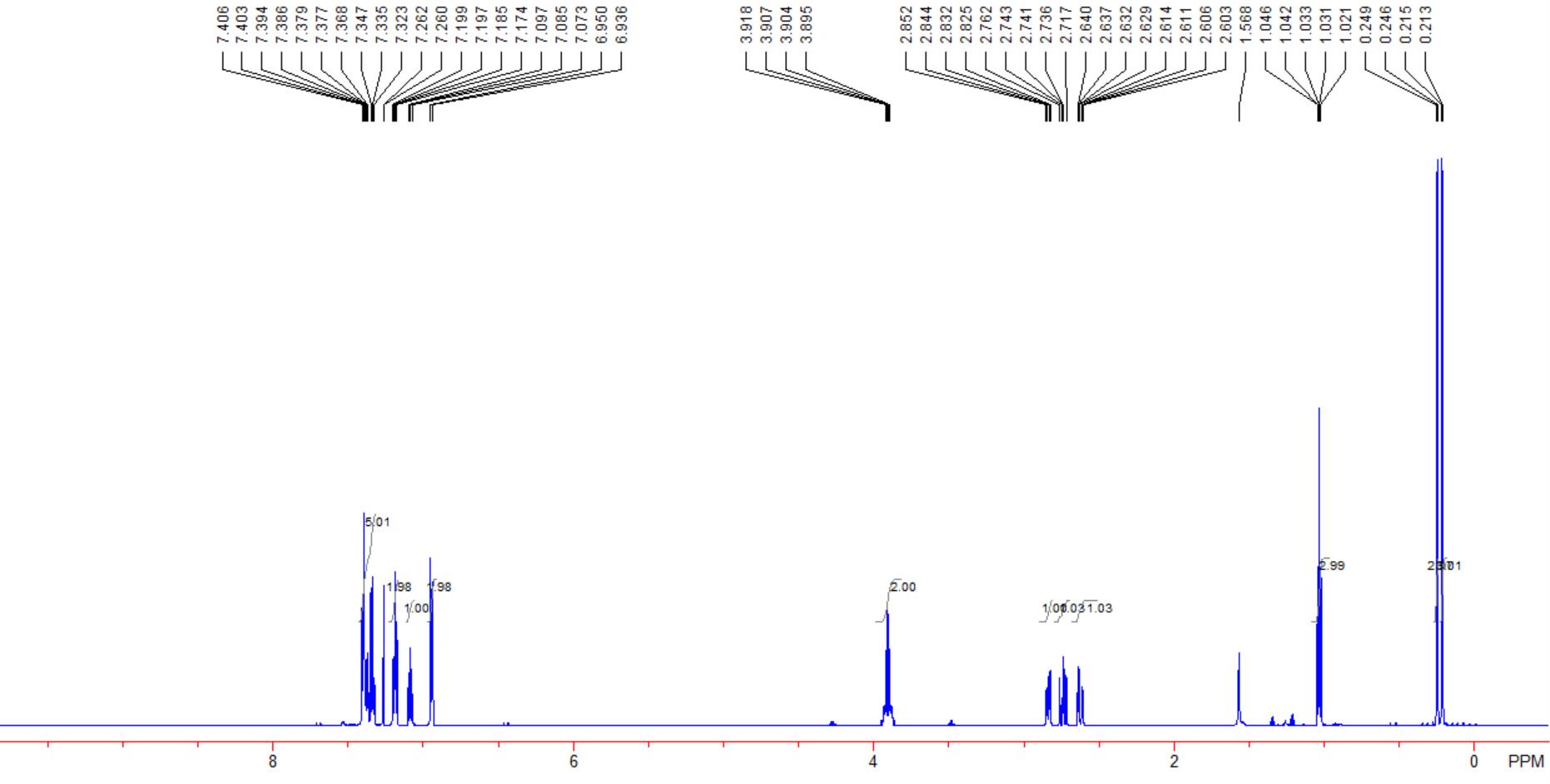


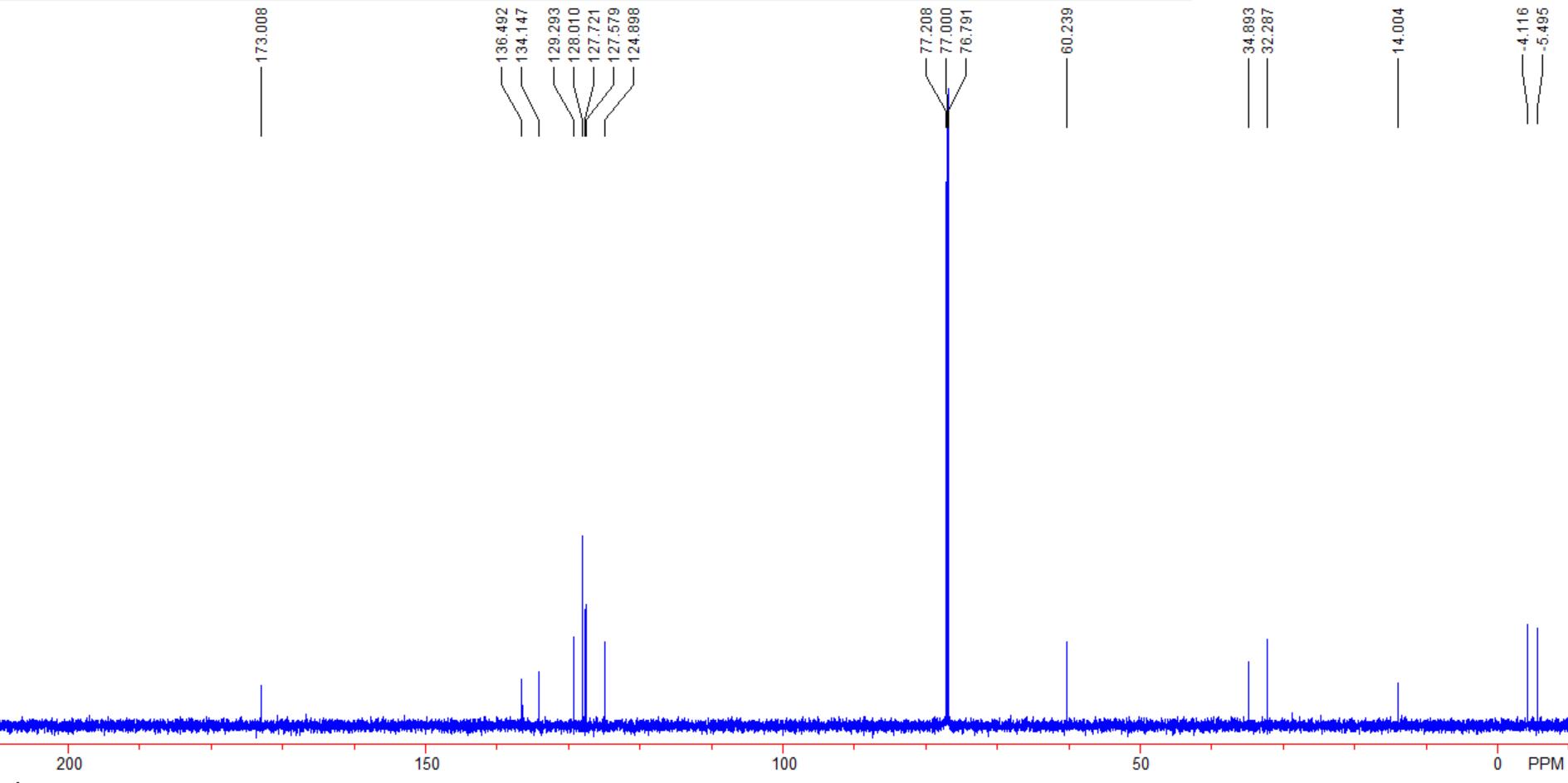

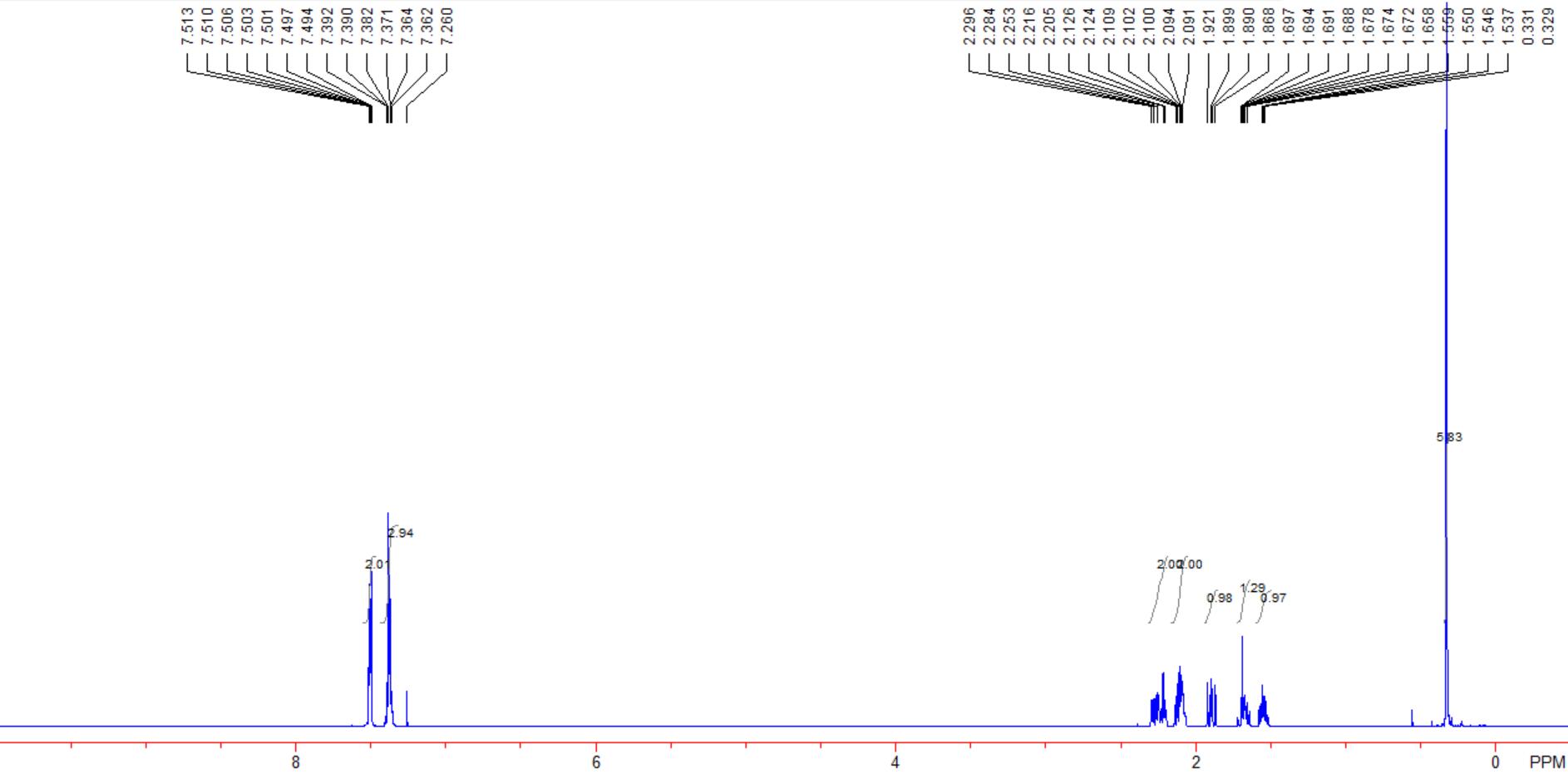


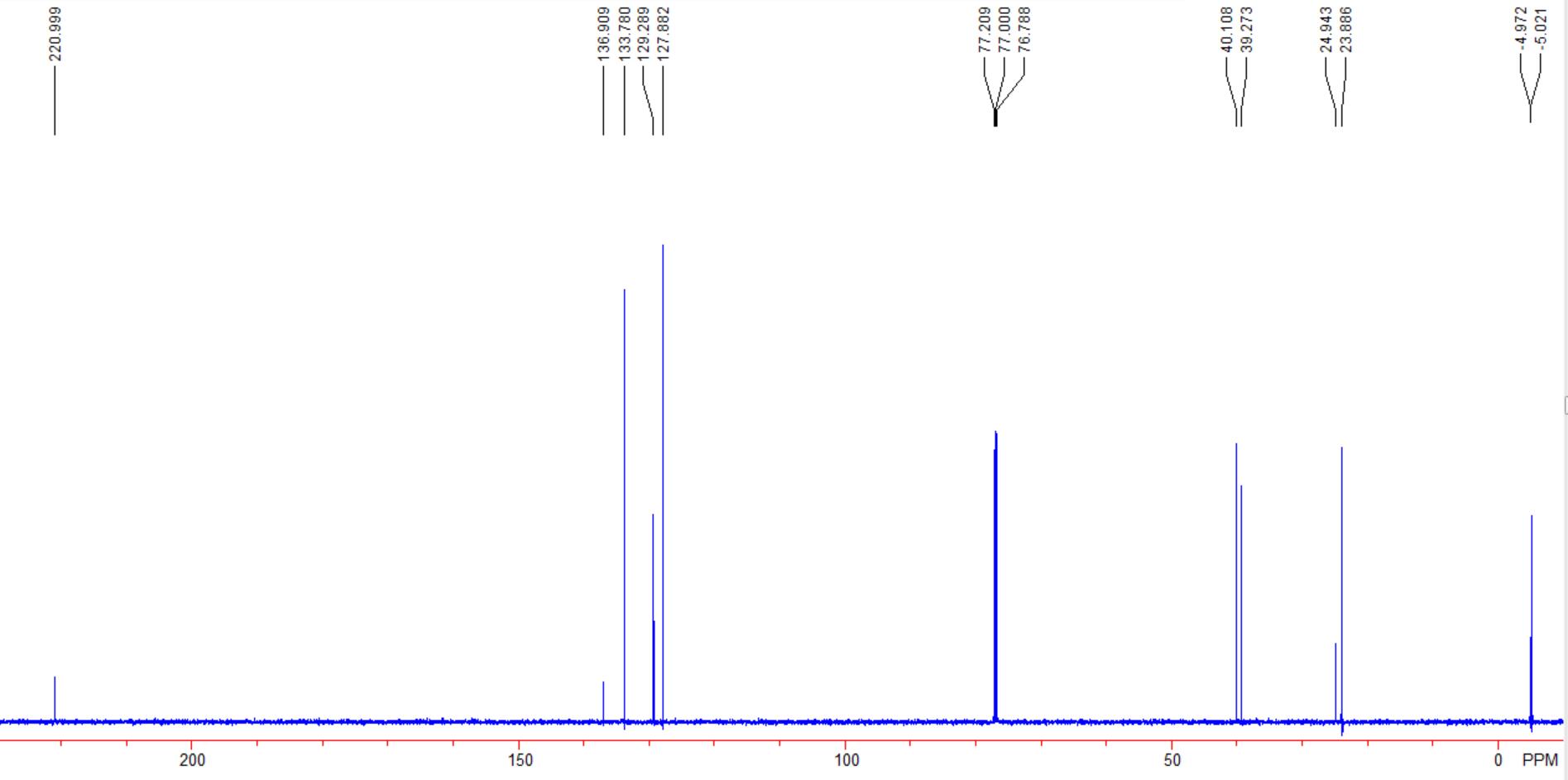

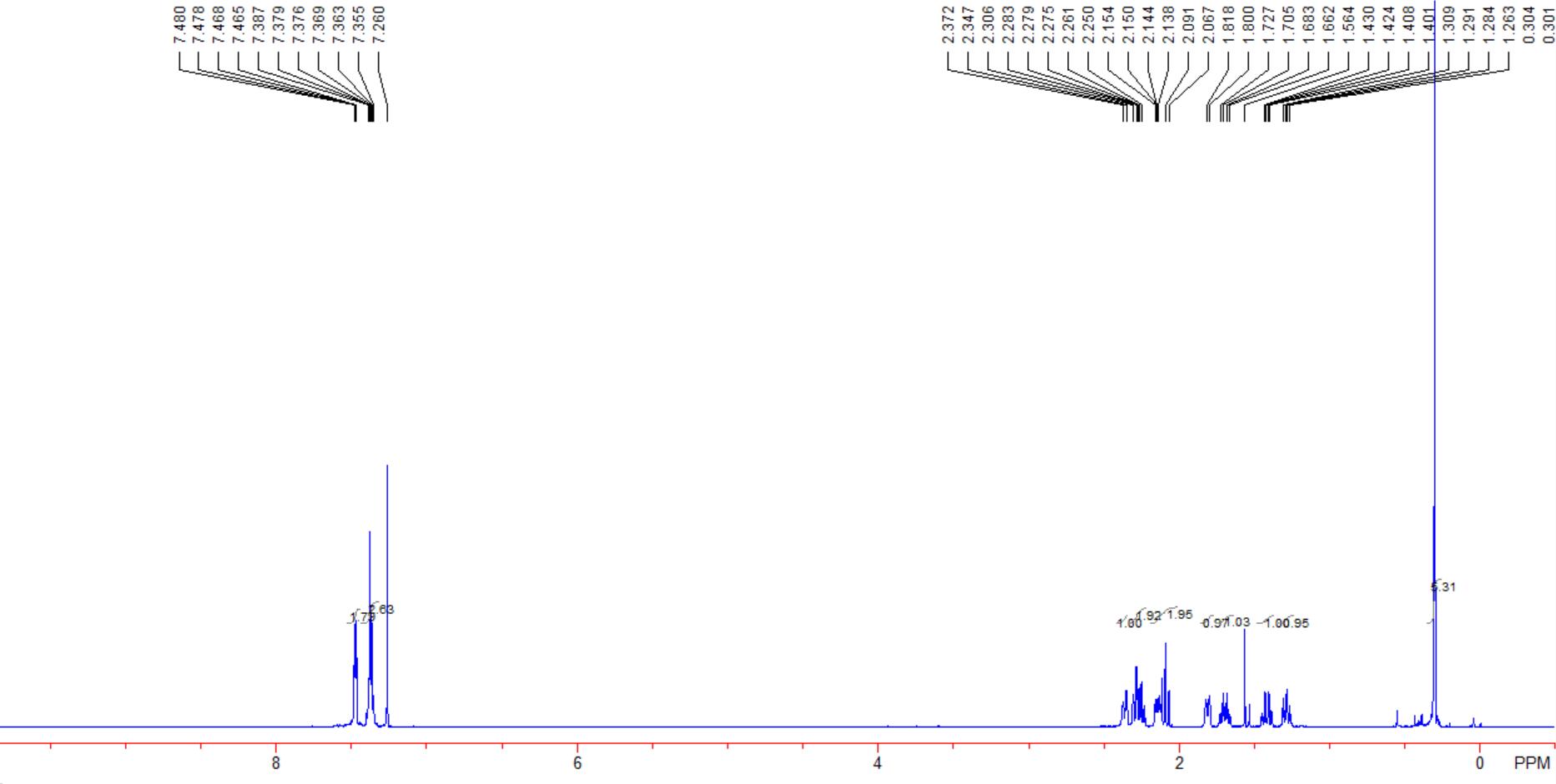


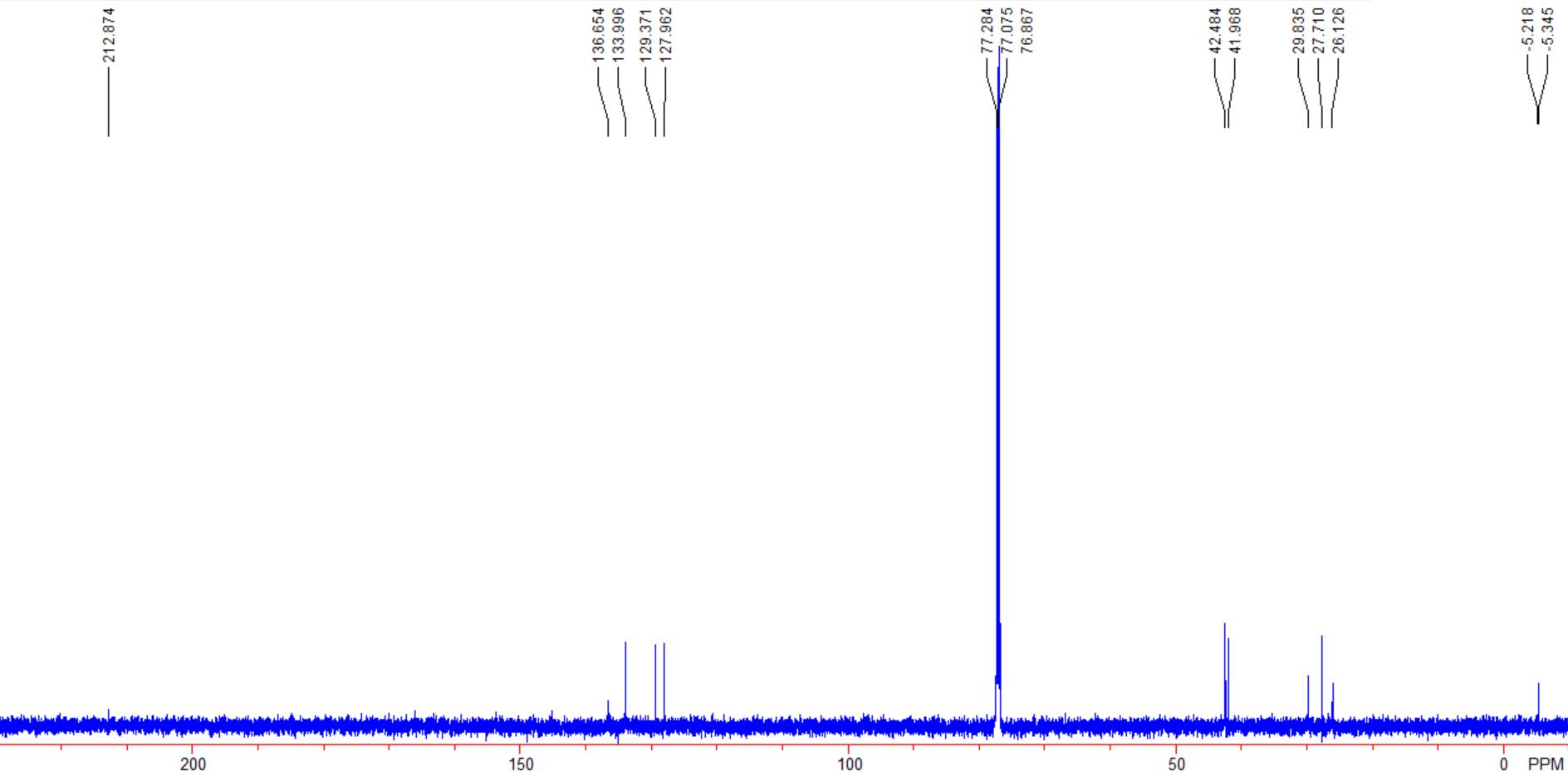

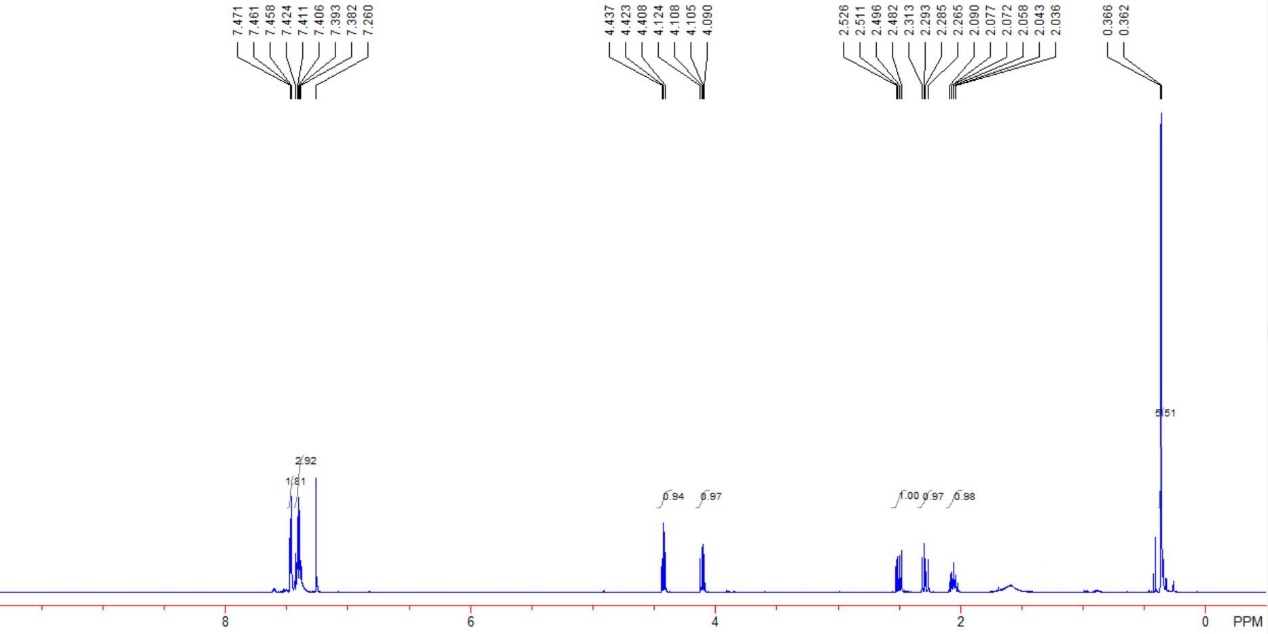


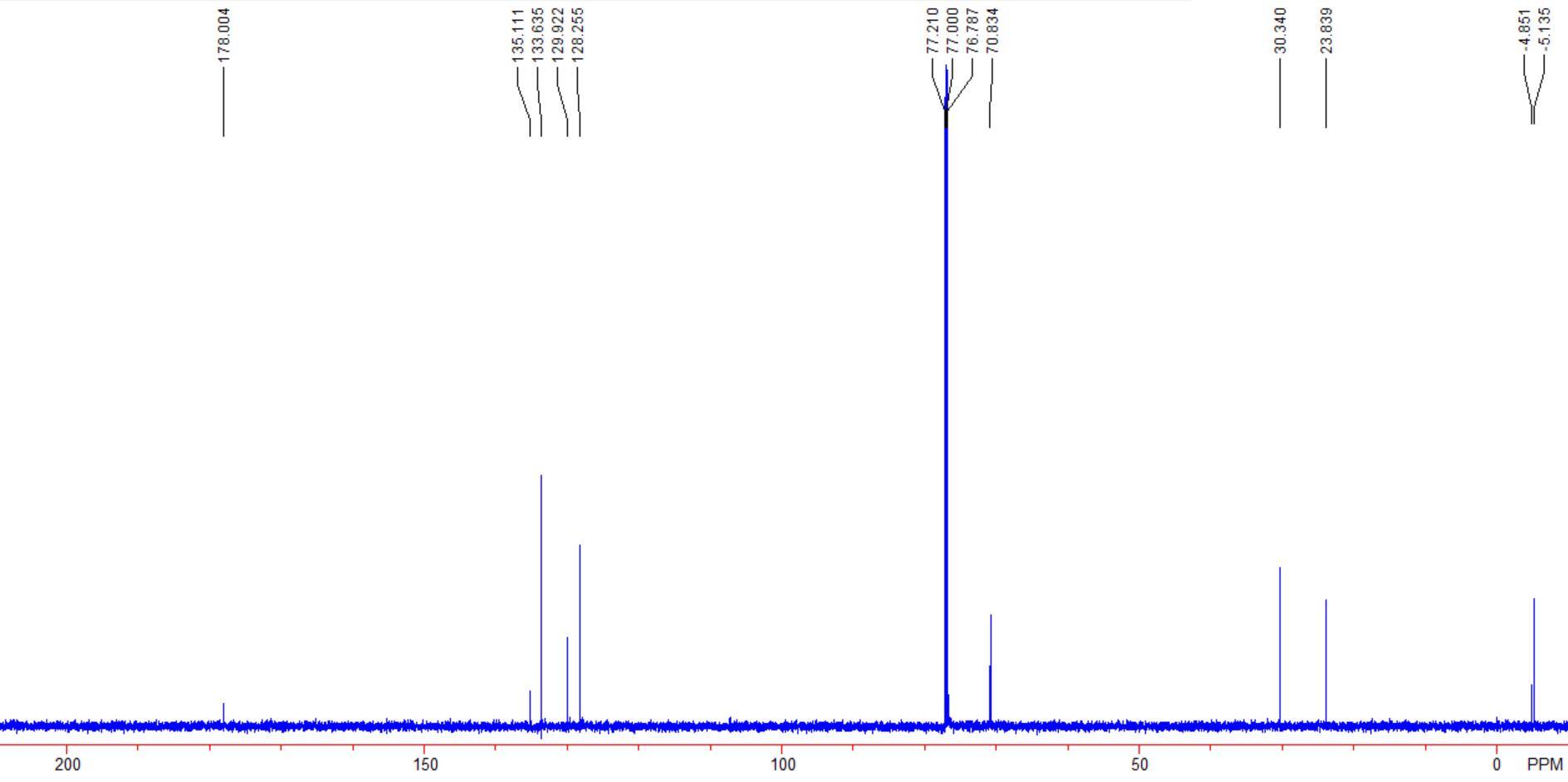

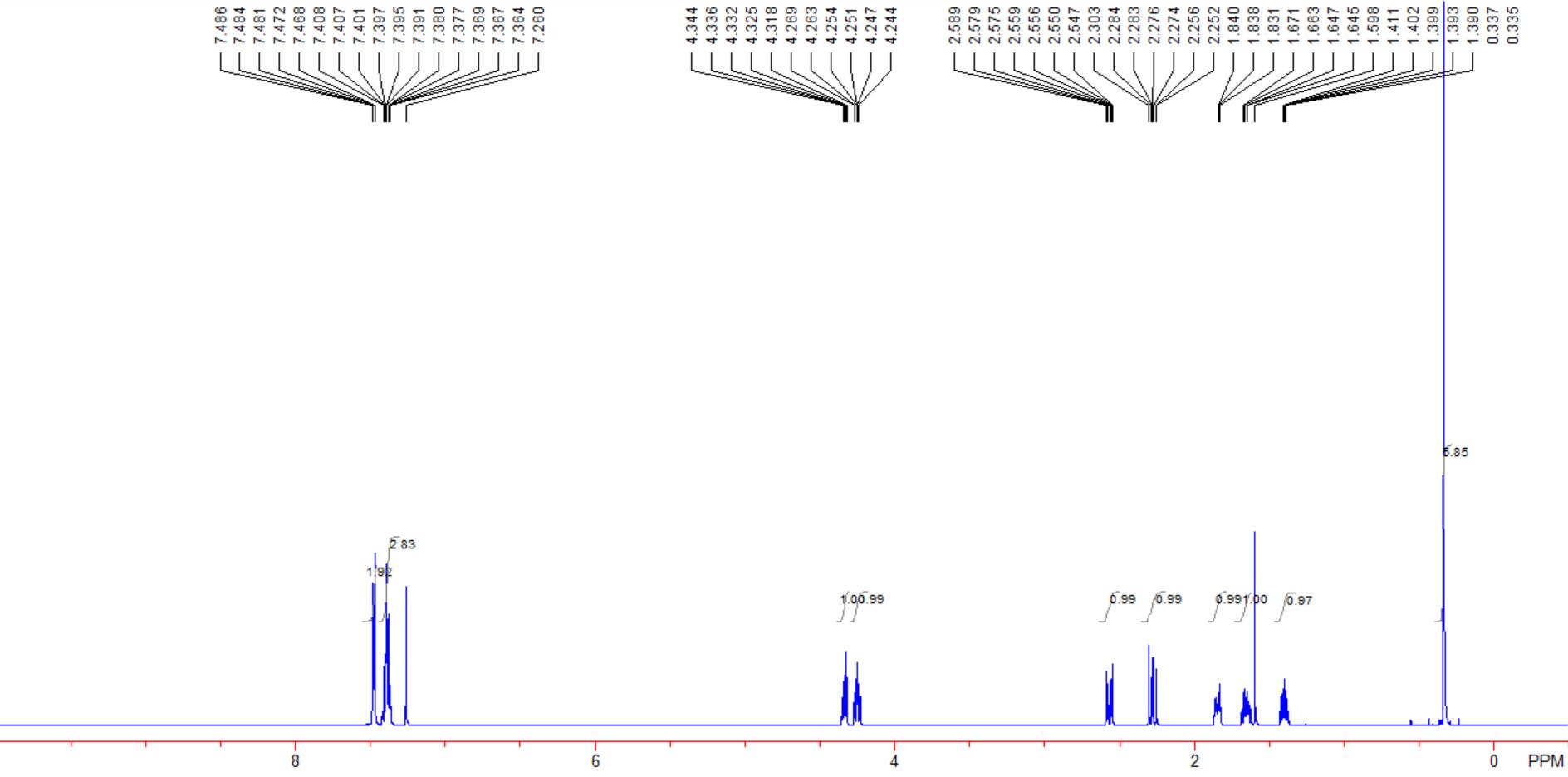


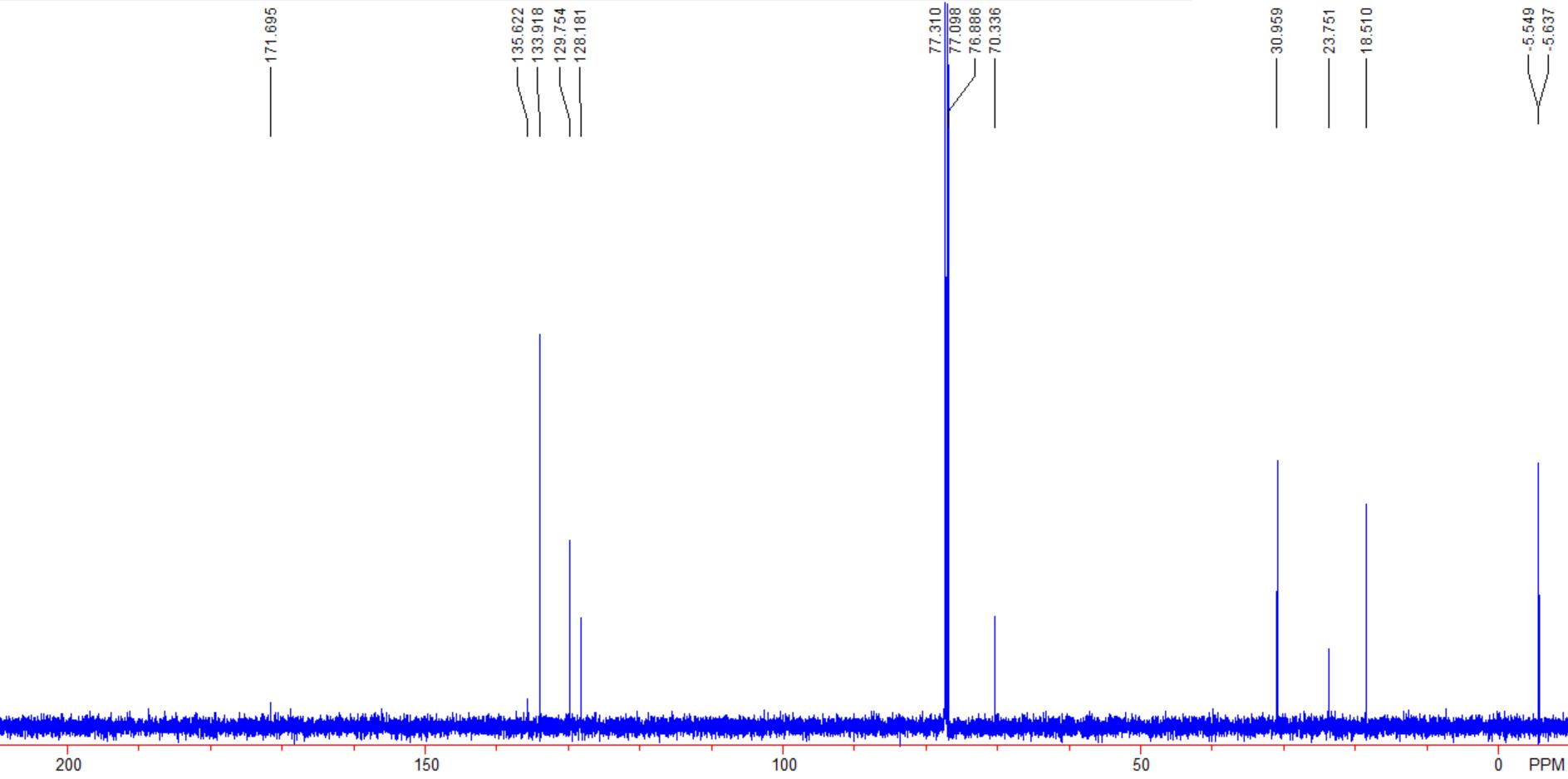

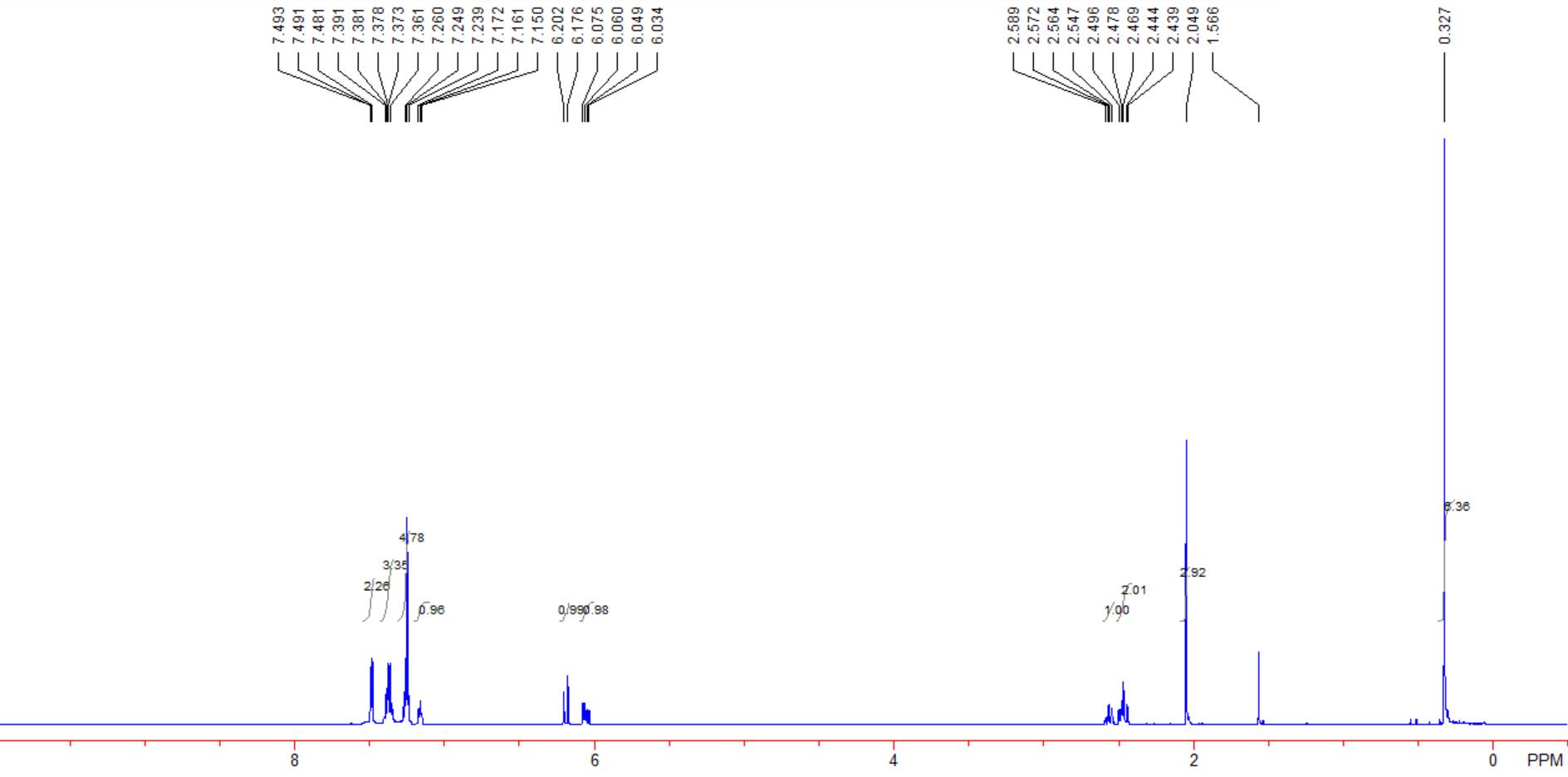


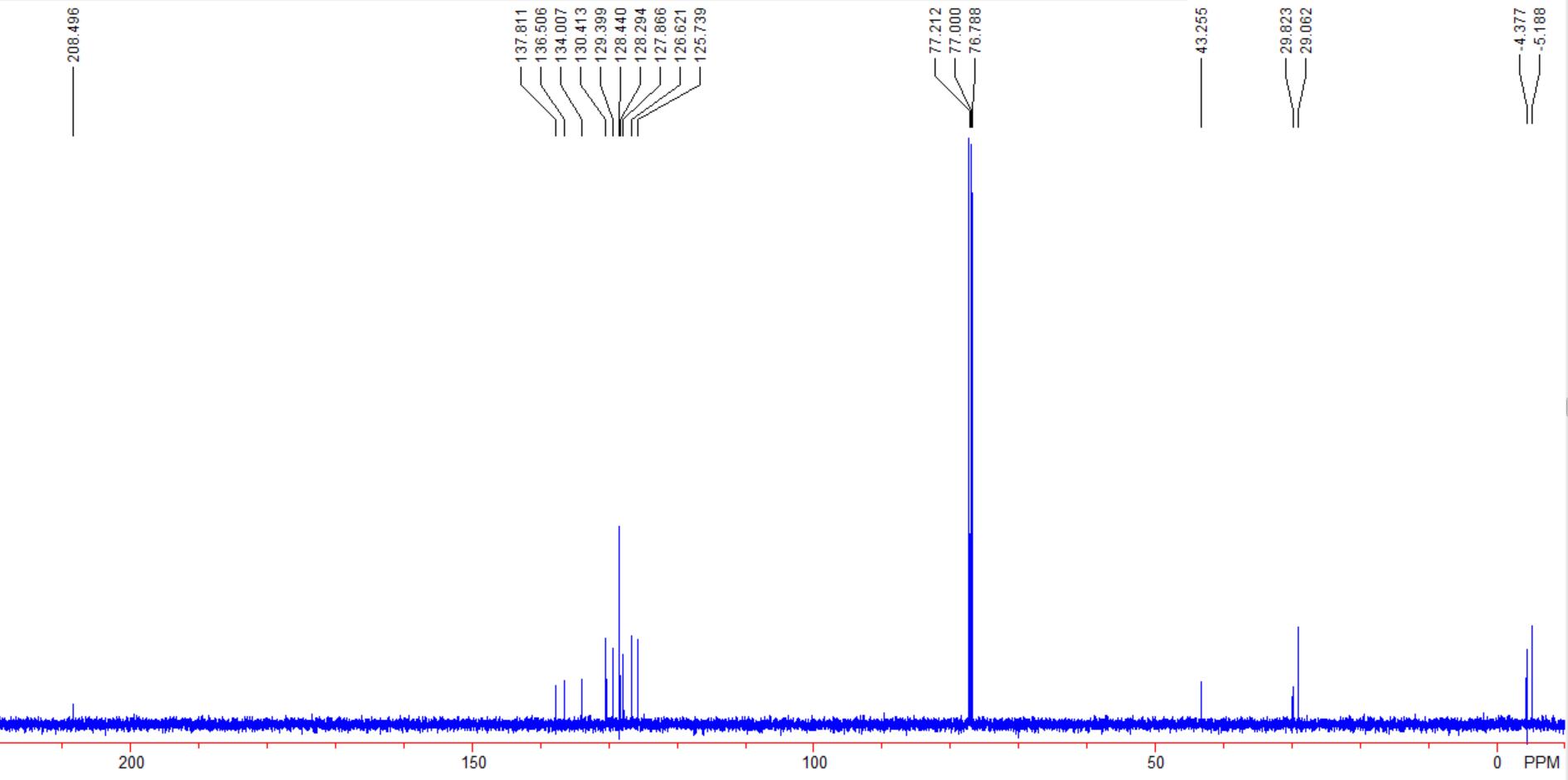

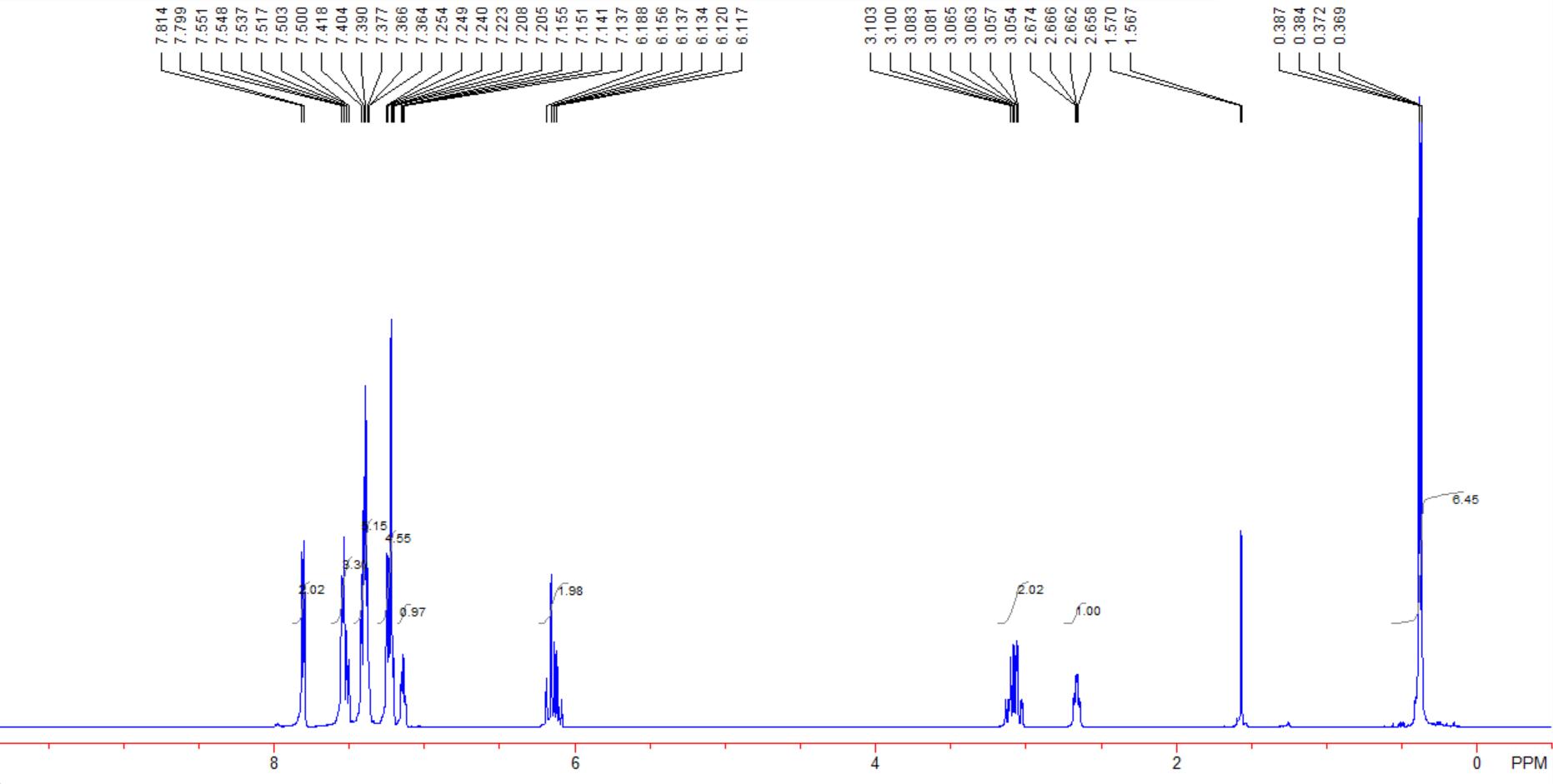


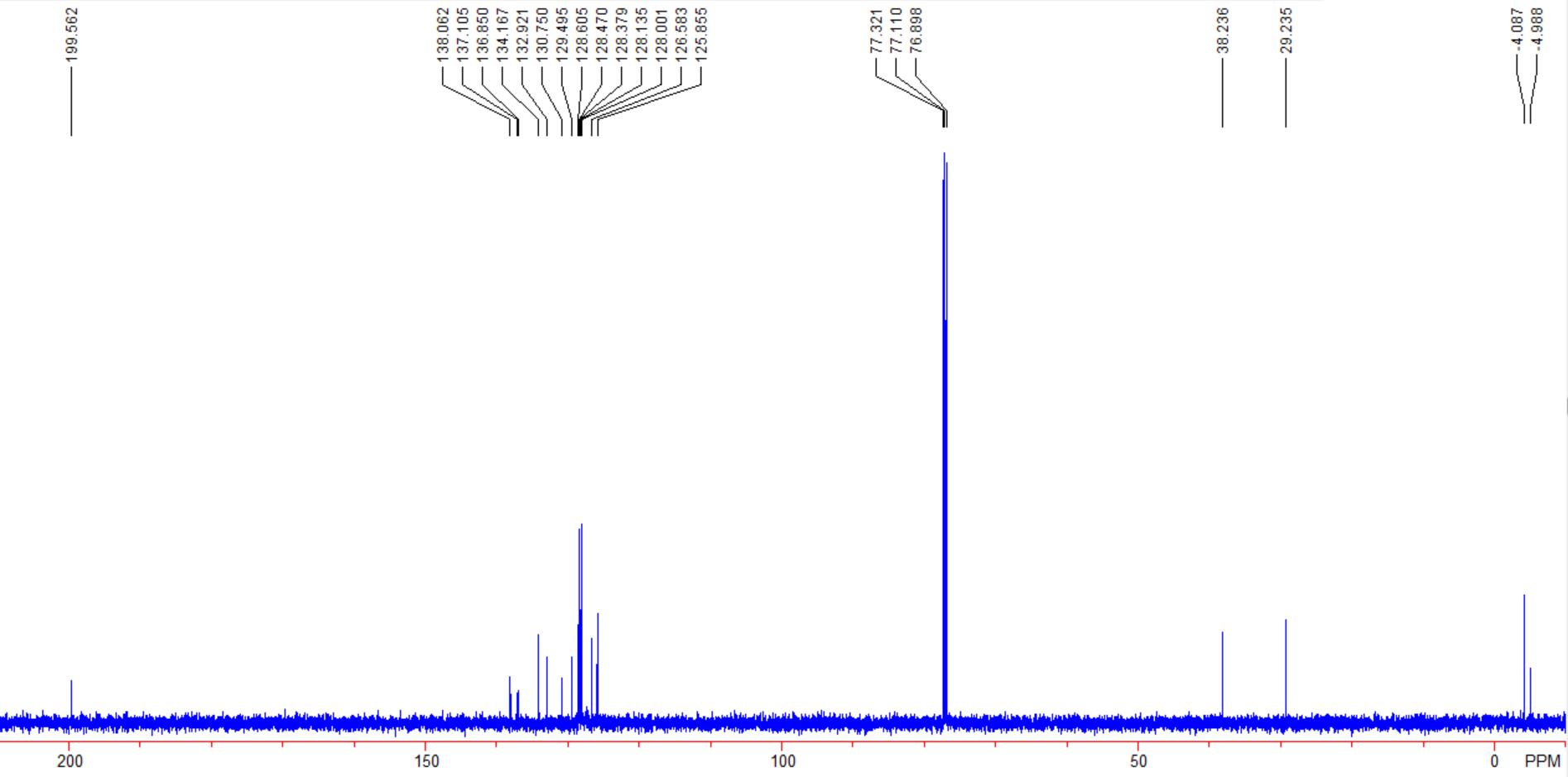

Supplement: Supplementary file 1 [file polymers-10-00385-s001.docx]
